# Assessing the Impacts of Cu and Mo Engineered Nanomaterials on Crop Plant Growth Using a Targeted Proteomics Approach

**DOI:** 10.1021/acsagscitech.3c00431

**Published:** 2023-12-22

**Authors:** Weiwei Li, Arturo A. Keller

**Affiliations:** Bren School of Environmental Science and Management, University of California at Santa Barbara, Santa Barbara, California 93106, United States

**Keywords:** engineered nanomaterials, root exposure, leaf exposure, targeted proteomics, liquid chromatography with tandem mass spectrometry

## Abstract

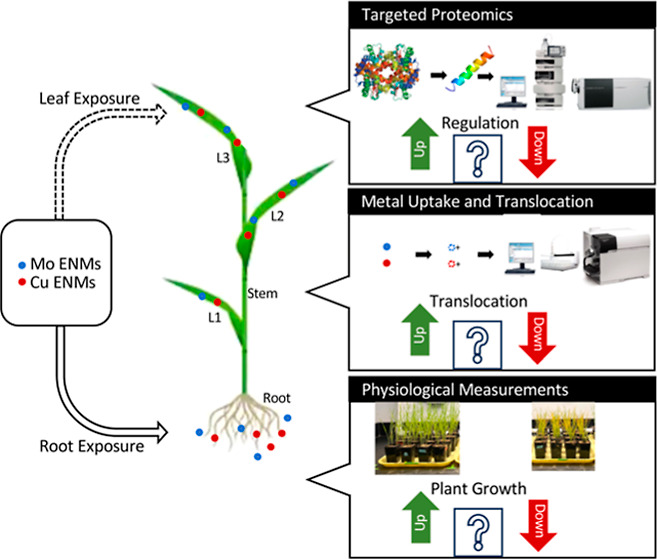

In this study, we investigated the effects of molybdenum (Mo)-based nanofertilizer and copper (Cu)-based nanopesticide exposure on wheat through a multifaceted approach, including physiological measurements, metal uptake and translocation analysis, and targeted proteomics analysis. Wheat plants were grown under a 16 h photoperiod (light intensity 150 μmol·m^–2^·s^–1^) for 4 weeks at 22 °C and 60% humidity with 6 different treatments, including control, Mo, and Cu exposure through root and leaf. The exposure dose was 6.25 mg of element per plant through either root or leaf. An additional low-dose (0.6 mg Mo/plant) treatment of Mo through root was added after phytotoxicity was observed. Using targeted proteomics approach, 24 proteins involved in 12 metabolomic pathways were quantitated to understand the regulation at the protein level. Mo exposure, particularly through root uptake, induced significant upregulation of 16 proteins associated with 11 metabolic pathways, with the fold change (FC) ranging from 1.28 to 2.81. Notably, a dose-dependent response of Mo exposure through the roots highlighted the delicate balance between nutrient stimulation and toxicity as a high Mo dose led to robust protein upregulation but also resulted in depressed physiological measurements, while a low Mo dose resulted in no depression of physiological measurements but downregulations of proteins, especially in the first leaf (0.23 < FC < 0.68) and stem (0.13 < FC < 0.68) tissues. Conversely, Cu exposure exhibited tissue-specific effects, with pronounced downregulation (18 proteins involved in 11 metabolic pathways) particularly in the first leaf tissues (root exposure: 0.35 < FC < 0.74; leaf exposure: 0.49 < FC < 0.72), which indicated the quick response of plants to Cu-induced stress in the early stage of exposure. By revealing the complexities of plants’ response to engineered nanomaterials at both physiological and molecular levels, this study provides insights for optimizing nutrient management practices in crop production and advancing toward sustainable agriculture.

## 1 Introduction

Engineered nanomaterials (ENMs) have gained attention in the field of agriculture, particularly as nanopesticides and nanofertilizers, with the aim of enhancing agricultural productivity and sustainability,^1,2^ to address the challenges of feeding a growing global population in the face of climate change. By minimizing the quantity of pesticides needed and providing more controlled release mechanisms, nanopesticides can offer more targeted and efficient delivery of active ingredients, promoting more environmentally friendly and sustainable agriculture.^3^ Similarly, nanofertilizers are designed to enhance nutrient availability to plants with their controlled release mechanisms to ensure that nutrients are available when needed and make agriculture more sustainable.^4^ However, the physicochemical properties of ENMs, such as small particle size and high surface area, may increase their toxicity potential.^3,4^ Thus, understanding how ENMs interact with plants is essential to ensure both enhanced productivity and minimal negative impacts on the environment and human health.

Omics technologies have revolutionized our ability to understand and analyze the complex molecular responses of plants to various environmental stressors, including ENMs.^5,6^ The omics approaches employed in plant stress mechanism responses research include genomics (gene level), transcriptomics (mRNA level), proteomics (protein level), and metabolomics (metabolite level).^7^ These approaches allow researchers to delve into different molecular layers to understand how plants react to stressors. Several studies have adopted nontargeted proteomics to investigate plant responses after exposure to nanoparticles (NPs) such as Ag-NP,^8,9^ Al_2_O_3_–NP, and Zn-NP.^9^ Responsive protein levels perturbed due to the exposure to ENMs are involved in biological pathways such as oxidative stress tolerance, electron transfer and signaling, transcription and protein degradation, nitrogen metabolism, oxidative stress regulation, photosynthesis, and protein biosynthesis and turnover.^8−12^ Although nontargeted proteomics is a useful tool to discover disturbed protein pathways, it has limited accuracy and reproducibility due to the characteristics of the full-spectrum scan.^13^ Targeted proteomics can add a layer of depth by directly analyzing the changes in the expression of specific proteins, with accuracy and reproducibility since it uses selected reaction monitoring and focuses on a defined set of proteins or peptides.^14−16^ However, the absence of targeted proteomics studies of plant responses to ENMs represents a notable gap in current knowledge. In addition, by employing advanced analytical techniques, researchers can move beyond static snapshots and delve deeper into the temporal aspects of molecular responses. This refined approach can enhance our understanding of complex biological processes, providing insights into the kinetics, dynamics, and adaptability of organisms in response to changing environmental or experimental conditions.

For this study, we considered wheat (*Triticum aestivum*), a crop of global importance, and the effect of two types of ENMs, Cu- and Mo-based. We selected 24 proteins based on previous studies that reported them to be more likely to be perturbed by the exposure to ENMs, and their signature peptides were selected based on a public wheat proteome database, as detailed in our previous study (Table 1).^17^ These targeted proteins are involved in several key metabolomic pathways, such as photosynthesis-related pathways (e.g., photorespiratory pathway and Calvin cycle) and respiration-related pathways (e.g., glycolysis, tricarboxylic acid (TCA) cycle, and mitochondrial electron transport). A study reported a significant increase in the expression of the light-harvesting complex II (LHCII) b gene in *Arabidopsis thaliana* when exposed to titanium dioxide NPs (TiO_2_-NPs).^18^ Another study observed that zinc oxide NPs (ZnO-NPs) improved antioxidant capacity and enhanced photosynthetic efficiency in tomato plants.^19^ This improvement in antioxidant mechanisms and photosynthesis could contribute to better plant growth and stress tolerance. In addition, nitrogen-cycle-related pathways, such as nitrogen metabolism and amino acid metabolism, were reported to promote productivity of cucumber due to the 51% more nitrogen accumulation from the application of TiO2-NPs.^20^ Another study indicated that the application of iron (Fe), cobalt (Co), and copper (Cu) NPs resulted in increased nitrogen accumulation and up to a 16% increase in crop yield in soybean plants.^21^ Moreover, tolerance-related pathways such as oxidative stress regulation were reported to strengthen abiotic stress resistance caused by ENMs in crop plants.^22^

**Table 1:** List of 24 Selected Targeted Proteins with Related Pathways and Signature Tryptic Peptides

pathway ID	pathway	protein ID	accession number	protein	signature peptide
A	amino acid metabolism	P1	AT3G23810	AA degradation methionine	LVGVSEETTTGVK
		P2	AT5G17920	AA synthesis methionine	GNATVPAMEMTK
		P3	AT1G02500	S-adenosylmethionine synthase	FVIGGPHGDAGLTGR
B	fermentation	P4	AT1G23800	aldehyde dehydrogenase	VAEGDAEDVDRAVVAAR
C	glycolysis	P5	AT2G36460	glycolysis cytosolic branch UGPase	FASINVENVEDNRR
		P6	AT5G17310	glycolysis cytosolic branch aldolase	VQLLEIAQVPDEHVNEFK
D	H+ transporting pyrophosphatase	P7	AT1G15690	transport H+ transporting pyrophosphatase	AAVIGDTIGDPLK
E	hormone metabolism	P8	AT1G55020	lipoxygenase	GMAVPDSSSPYGVR
F	mitochondrial electron transport/ATP synthesis	P9	AT1G78900	transport p- and v-ATPase	SGDVYIPR
		P10	AT4G09650	ATP synthase delta chain	TALIDEIAK
		P11	AT5G08670	ATP synthase beta subunit	IGLFGGAGVGK
		P12	AT2G07698	ATP synthase F1-ATPase	TAIAIDTILNQK
G	nitrogen metabolism	P13	AT5G07440	glutamate dehydrogenase	TAVAAVPYGGAK
		P14	AT5G04140	glutamate synthase ferredoxin-dependent	IGGLTLNELGR
H	photorespiratory pathway	P15	AT1G70580	peroxisomal aminotransferases	KALDYEELNENVK
		P16	AT5G23120	photosystem II stability/assembly factor HCF136	AADNIPGNLYSVK
I	photosynthesis/Calvin cycle	P17	AT2G21330	Calvin cycle aldolase	TVVSIPNGPSELAVK
		P18	AT3G54050	Calvin cycle FBPase	YIGSLVGDFHR
		P19	AT2G36460	fructose-bisphosphate aldolase	VAPEVIAEYTVR
		P20	AT3G26650	Calvin cycle GAP	TLAEEVNQAFR
J	redox	P21	AT1G20620	catalase	TWPEDVVPLQPVGR
K	TCA/org transformation	P22	AT5G43330	malate dehydrogenase	EFAPSIPEK
		*P*23	AT4G35830	TCA aconitase	VAEFSFR
L	tetrapyrrole biosynthesis	P24	AT5G08280	tetrapyrrole synthesis porphobilinogen deaminase	TLGELPAGSVIGSASLRR

A Cu-based nanopesticide (Cu(OH)_2_-NMs) and Mo-based nanofertilizer (MoO_3_–NMs) were selected as ENM treatments to wheat plants. To determine a realistic yet impactful experimental dose, the recommended field application doses, previous studies, and the potential for eliciting significant plant responses were considered. According to the Fertilizer Institute (tfi.org), the recommendation for field application of Cu and Mo is 3–16 kg/hectare and 0.6–2 kg/hectare, respectively, which is 0.8–5 mg Cu/plant and 0.2–0.6 mg Mo/plant based on a wheat population of 3.2–3.7 million plants/hectare. The application dose will also support the nutritional requirements of Cu (5 ppm)^23^ and Mo (0.1 ppm),^24^ which are essential micronutrients for plants. Previous ENM-related metabolomics studies revealed significant alterations of metabolites at 12 mg Cu/plant exposure dose for spinach,^25^ 6.7 mg Cu/plant exposure dose for cucumber,^26^ 6 mg Cu/plant exposure dose for soybean,^27^ and 8 mg Mo/plant exposure dose for corn and wheat (no significance with a lower dose of 1.6 mg Mo/plant for wheat).^28^ Considering both recommended field application doses and previous studies, we initially chose 6.25 mg of element/plant for both Cu and Mo. Then, the recommended dose for field application of Mo (0.6 mg of Mo/plant) was added to the experiment. Two different exposure techniques were studied, root and leaf exposure. For each exposure approach, 3 treatment groups were considered, including the control group, Cu exposure group, and Mo exposure group.

This study aims to address this gap by pioneering the application of targeted proteomics for investigating plant response to these micronutrients in nanoscale form to provide focused and precise insights into the specific proteins and pathways impacted. The study will also shed light on the potential applications and risks associated with using these nanomaterials in agriculture, offering valuable insights for optimizing nutrient supplementation strategies and minimizing adverse effects on plant growth.

## 2 Materials and Methods

### 2.1 Materials

Cu(OH)_2_-NMs (99.5% purity, US3078) and MoO_3_–NMs (99.94% purity, US3330) were purchased from U.S. Research Nanomaterials Inc. (Houston, TX, USA). *T. aestivum* (wheat) seeds were purchased from Harmony Farms KS (Jennings, KS, USA). The reagents used during sample processing, such as sodium hypochlorite solution, Triton X-100, dithiothreitol (DTT), iodoacetamide (IAA), trypsin protease, trifluoroethanol (TFE), protease inhibitors cocktail, formic acid, ammonium acetate, trichloroacetic acid (TCA), dimethyl sulfoxide (DMSO), 0.5 M pH 8.0 ethylenediaminetetraacetic acid (EDTA), sucrose, high-performance liquid chromatography (HPLC)-grade water, methanol, acetone, and isopropyl alcohol (IPA), were purchased from Sigma-Aldrich (St. Louis, MO, USA). Urea, ammonium bicarbonate, and acetonitrile (ACN) were obtained from Spectrum Chemicals (New Brunswick, NJ, USA). Other reagents including Tris-buffered phenol solution, 1.5 M pH 8.8 Tris–HCl solution, LysC/trypsin protease mix, phenylmethanesulfonyl fluoride (PMSF), 2-mercaptoethanol (2 ME), sodium *n*-dodecyl sulfate (SDS), and materials such as 5 and 15 mL Eppendorf centrifuge tubes were purchased from Fisher Scientific (Waltham, MA, USA). C-18 cartridges (Waters Sep-Pak C18 1 cc, 50 mg of sorbent) were purchased from Waters Corporation (Milford, MA, USA). The analytical standards of the 24 selected peptides (Table 1) and 1 isotopic labeled peptide standard to use as internal standard, including SVHEPMQTGLK{Lys(13C6,15N2)}, SGDVYIPR{Arg(13C6,15N4)}, TALIDEIAK{Lys(13C6,15N2)}, and KPWNLSFSFGR{Arg(13C6,15N4)}, were purchased from GenScript (Piscataway NJ, USA). These standards were synthesized as ordered in the white lyophilized powder phase with ≥95% HPLC purity.

### 2.2 Wheat Growth and Exposure Conditions

**Figure 1:**
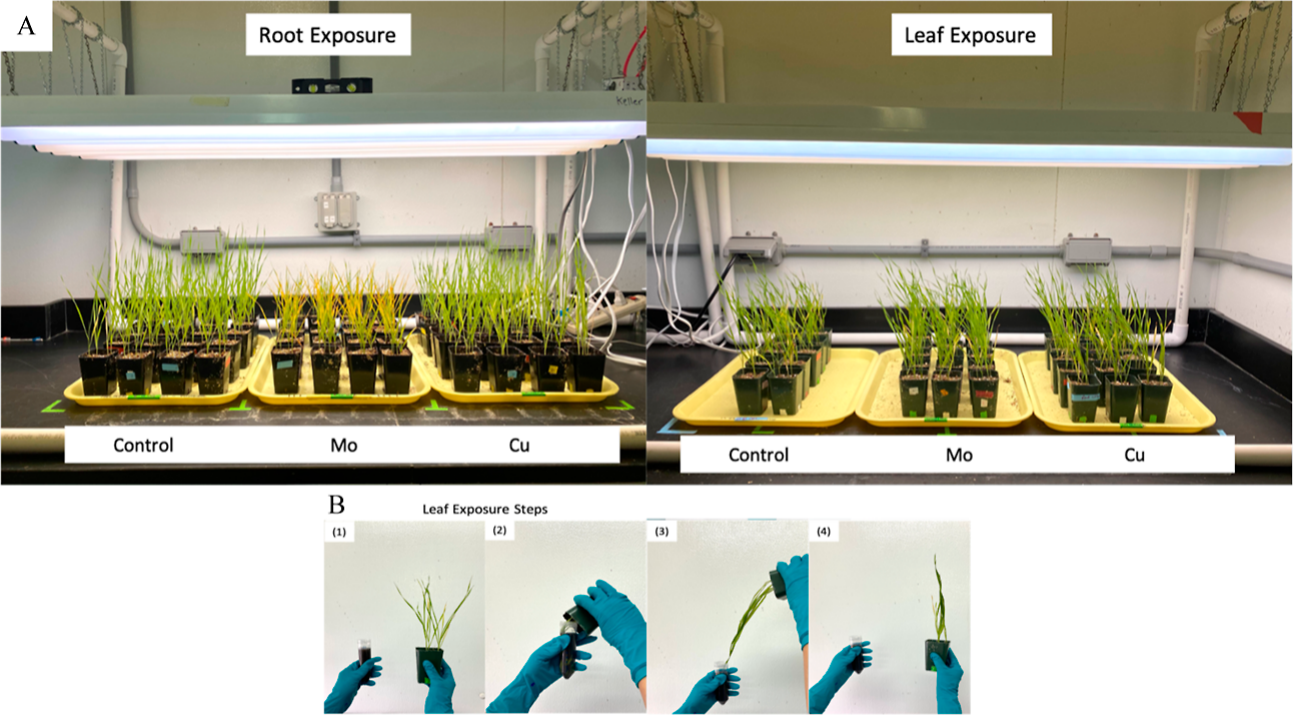
Wheat plant growth and exposure. (A) Images of plant growth with two different exposure techniques (root exposure and leaf exposure) and 3 treatment groups [control group, Mo exposure group (6.25 mg Mo/plant), and Cu exposure group (6.25 mg Cu/plant)]; (B) Leaf exposure steps: (1) prepare ENM suspensions in a 50 mL centrifuge tube; (2) insert all the leaves into the tube, swirling the leaves gently and soaking the leaves in solution for 10 s; (3) remove the leaves and let them dry for 10 s; (4) bring plant upright and let it dry for 15 min and then repeat steps 2–4 for another 2 times for a total of 3 daily exposures.

Wheat seeds were placed in 6 groups according to treatments and exposure methods (Figure 1A), including root exposure control, Cu exposure through root, Mo exposure through root, leaf exposure control, Cu exposure through leaf, and Mo exposure through leaf. First, all wheat seeds were sterilized in 1% sodium hypochlorite solution for 10 min and then rinsed for 5 times with NANOpure water, followed by soaking in NANOpure water overnight for germination. Then, the germinated seeds were planted into vermiculite saturated with 10% Hoagland solution with 4 seeds per pot following the same procedure as in previous studies.^17^ Plants were grown under a 16 h photoperiod (light intensity 150 μmol·m^–2^·s^–1^) for 4 weeks at 22 °C and 60% humidity and watered with 20 mL of diluted 10% Hoagland solution daily to maintain a 70–90% water content and provide sufficient nutrients for plant growth.^17,28^

For root exposure groups, ENM suspensions were prepared in 10% Hoagland solution at 1250 mg of Cu or Mo element per liter. On day 7, in contrast to watering with 20 mL of 10% Hoagland water for the root exposure control group, the Cu and Mo exposure groups were watered with 20 mL of ENM suspensions. At the 4 seedling locations, 5 mL of the ENM suspensions was added to the pots with a 5 mL pipet to ensure even exposure, for a total of 20 mL/pot. The total amount of ENM exposure is 25 mg of Cu or Mo per pot, which is 6.25 mg of element per plant. For leaf exposure groups, the surfactant (Triton X-100, BioXtra, p/n: T9284) was employed to improve the wettability of leaf surfaces and prevent off-target drift.^29^ ENM suspensions were prepared with 500 mg of Cu or Mo element per liter of the surfactant solution (0.2% Triton X-100 in NANOpure water). From day 22 to day 28, plant leaves were soaked 3 times per day in 50 mL centrifuge tubes with freshly prepared ENM suspensions for exposure groups, or in the surfactant solution for leaf control group (Figure 1B). The amount of applied ENM suspensions was calculated by considering the weight of solutions measured before and after leaf soaking and the concentration of solution. On average, the daily exposure volume for both Cu and Mo suspensions was around 7 mL. After 7 days of leaf exposure, the total amount of ENM exposure was 25 mg of Cu or Mo per pot, which is 6.25 mg of element per plant as well. For both exposure approaches, at least 40 plant replicates (in 10 pots and 4 plants per pot) were grown for each treatment.

After the initial studies with 6.25 mg of Mo/plant, it became clear that the excessive concentration of Mo had a negative physiological effect, particularly when exposed to Mo ENMs via roots. To further study the dose effect of Mo exposure through the roots, a lower concentration of 0.6 mg Mo/plant via the roots, which is the recommended dose for field application of Mo, was added to the experiment.

### 2.3 Wheat Harvesting, Physiological Measurements, and Tissue Homogenization

After 28 days, the plants were harvested and grouped into the 6 treatments followed by rinsing with NANOpure water. Physiological measurements, including leaf color, biomass, and length of the shoot (tissues above the soil) and root parts, were recorded for each group. Three leaves emerged from each plant during the 4 week growth period. The harvested leaves were labeled as leaf #1 (L1), leaf #2 (L2), and leaf #3 (L3) with L1 being the first leaf to emerge and L3 being the third leaf to emerge. To calculate the biomass distribution, the biomass was also measured for L1, L2, L3, and stem and root parts separately after cutting the plants into these five parts. After measurements, each of the 5 tissues from each treatment group was pooled and ground using a mortar and pestle with liquid nitrogen added for homogenization. The homogenized plant tissues (5 tissues ×6 treatment groups = 30 tissue samples) were stored in 50 mL centrifuge tubes at −80 °C until analyzed.

### 2.4 Metal Uptake and Translocation Analysis

In a previous study, we determined the dissolution rate of Cu- and Mo-based ENMs.^30^ The dissolution of Cu ENMs was relatively slow in both deionized (DI) water and root exudate solution, around 1% after 6 days and a rate of 0.001% per hour. In contrast, Mo ENMs dissolve relatively fast when placed in either DI water or root exudate solution, releasing around 31–35% of Mo ions within the first 6 h and 0.026–0.047% per hour thereafter. Thus, the wheat plants exposed via roots to Mo ENMs will also be exposed to a substantial amount of Mo^6+^, and even those exposed via the leaves would be exposed to released Mo ions. In contrast, the plants exposed to Cu ENMs would be exposed to low concentrations of Cu^2+^, in either exposure path.

To reveal the effect on metal element accumulation and distribution caused by ENM exposure during growth, the concentration of elements including Cu, Mo, and other nutrient elements such as K, Mg, Ca, P, Mn, Fe, and Zn in plant tissues was quantified via inductively coupled plasma–mass spectrometry (ICP–MS) analysis (Agilent 7900, Agilent Technologies). A 100 mg sample of the homogenized plant was weighed into a 50 mL digestion tube and mixed with 2 mL of PlasmaPure HNO_3_ (trace metals equal to or less than 1 ppb). Then, the tubes were covered with watch glasses and placed into a hot block digestion system (DigiPREP MS, SCP Science) to heat for 20 min at 115 °C, followed by the addition of 8 mL of H_2_O_2_ to continue to heat for 60 min at 115 °C. The digested solution was diluted to a total volume of 50 mL with NANOpure water. Finally, 4 mL of diluted digests was transferred into a 15 mL metal-free centrifuge tube and mixed with 4 mL of NANOpure water for the final dilution to ensure <2% acid content for ICP–MS analysis. Six points of calibration standards ranging from 1 to 1000 ppb were prepared for each analyzed element for quantification. For quality assurance/quality control (QA/QC) purposes, a midlevel of calibration standards followed by a solvent blank were injected after every 6 sample injections, and the recovery for QC injections was all within 80%–120%. The ICP–MS results were adjusted by the dilution factors.

### 2.5 Protein Extraction and Targeted Proteomics Analysis

To measure the concentration of the selected proteins, plant tissues were processed through protein extraction and precipitation, proteolytic digestion, and peptide purification before analysis using an Agilent 6470 triple quadrupole mass spectrometer coupled with an Agilent InfinityLab 1290 Infinity II Series liquid chromatography system.^17^ Three replicates were prepared for each sample. First, samples were processed using the optimized phenol extraction method from our previous study.^17^ Generally, 200 mg of plant tissue sample was extracted using a phenol extraction buffer and then partitioned with phenol solution (tris-buffered). Then, ice-cold 0.1 M ammonium acetate in methanol was mixed with phenol extracts and stored overnight at −20 °C for protein precipitation. The protein pellet was washed with 0.1 M ammonium acetate in methanol followed by 80% (v/v) acetone in DI water to remove phenol, methanol, and ammonium acetate, followed by solubilization with 8 M urea and 50 mM ammonium bicarbonate solution. Then, the protein in solution was reduced and alkylated with 5 mM DTT and 20 mM IAA followed by peptide digestion with 2 μg of trypsin enzyme overnight at 37 °C with rotation. Finally, the digested peptides were purified via solid-phase extraction (SPE) with a C-18 cartridge (Waters Sep-Pak C18 1 cc, 50 mg sorbent). The samples were reconstituted to 30% ACN in water with 5% formic acid and 3% DMSO for liquid chromatography with tandem MS (LC–MS/MS) analysis.

An Agilent Polaris 3 C18-Ether column (150 × 3.0 mm, p/n: A2021150 × 030) coupled with a gradient mobile phase [A: water + 0.1% (v/v) formic acid + 3% (v/v) DMSO; B: ACN + 0.1% (v/v) formic acid + 3% (v/v) DMSO] was used to analyze the peptides in the processed samples.^17^ The HPLC conditions and MS conditions are detailed in the Supporting Information (SI, Table S1). The total run time for each sample was 14 min, and a needle wash with TFE was done between injections to reduce carryover. The transitions and limit of detection (LOD) for each peptide can be found in Table S2. Eight levels of calibration standards ranging from 1 ng/mL to 100 ng/mL with 50 ng/mL of internal standards were prepared for quantitation.^17^ For QA/QC purpose, a midlevel of calibration standards followed by a solvent blank were injected after every 6 sample injections, and the recovery for QC injections was all within 80–120%.

### 2.6 Statistical Analysis

Box-and-whisker plots coupled with one-way analysis of variance (ANOVA) followed by *t*-test were used to compare the physiology measurements across different treatment groups with a significant threshold (*p*-value) at 0.05. Heatmaps were used to visually represent the patterns of metal uptake and transport for Cu, Mo, and other nutrient elements. A heatmap of protein abundance across different treatments also helped identify clusters of proteins with similar expression profiles and highlight differences or trends between the experimental groups. Partial least squares—discriminant analysis (PLS-DA) was conducted to visualize the separation between different treatment groups.^31^ Volcano plots were used to depict fold changes (FCs) versus statistical significance (negative logarithm of *p*-values), which helped highlight proteins with significant changes in expression. Then, FC bar plots were generated to prioritize proteins that exhibit substantial changes with magnitudes larger than 1.25-fold or smaller than 0.75-fold. In addition, Venn diagrams were used to visualize the overlaps and differences between different treatment groups and help identify common or unique proteins that are significantly affected by ENMs.

## 3 Results and Discussion

### 3.1 Physiology Measurements

**Figure 2:**
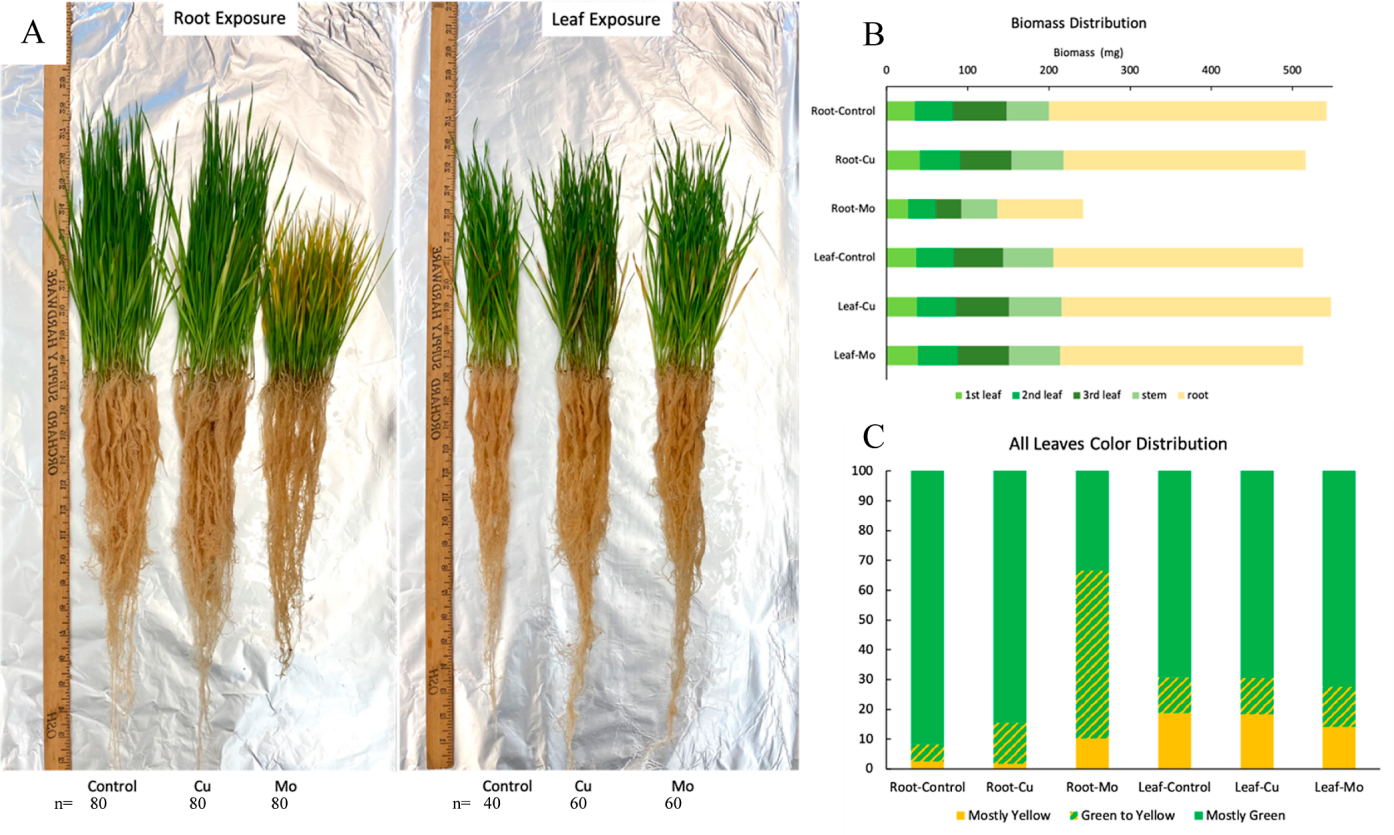
Wheat plant harvest. (A) Plants after harvest and wash [from left to right: root exposure control, Cu exposure through root (6.25 mg of Cu/plant), Mo exposure through root (6.25 mg of Mo/plant), leaf exposure control, Cu exposure through leaf (6.25 mg of Cu/plant), and Mo exposure through leaf (6.25 mg of Mo/plant)]; (B) biomass distribution of 6 groups; (C) leaves’ color distribution of 6 groups.

Plants were grouped into 6 treatments after harvest and washing (Figure 2A). Among all 6 groups, the Mo exposure through the root group (Root-Mo) was particularly distinct in its response to the ENMs. The Root-Mo group exhibited smaller plant mass and especially less root mass compared to that of all other groups (Figure 2B), which suggests that the Mo exposure through roots has a substantial impact on plant growth and development. In addition, the Root-Mo group produced the most yellow leaves, while the root exposure control group (Root-Control) produced the least yellow leaves (Figure 2C). The leaf color changes indicated the changes in photosynthetic efficiency and overall plant health. Additionally, the control of leaf exposure group (Leaf-Control) produced more yellow leaves than that of the Root-control, likely due to the usage of Triton X-100 as the surfactant for the leaf treatments, which suggests the potential interactions between surfactants and plant physiology.^29^

**Figure 3:**
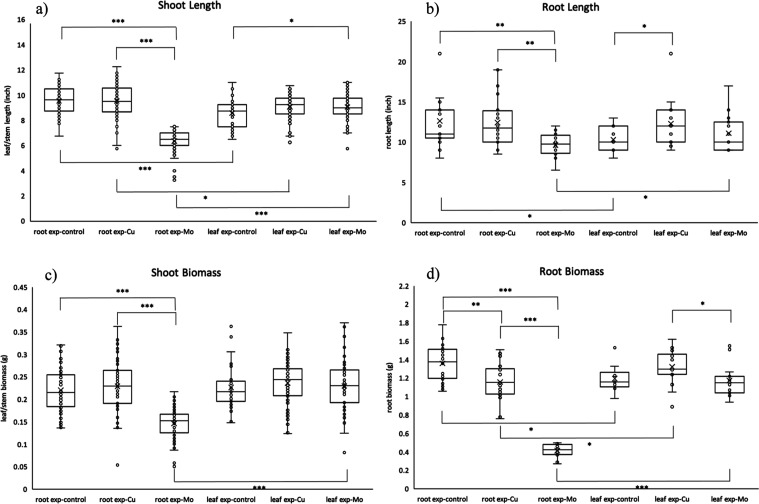
Box-and-whisker plot of (a) shoot length, (b) root length, (c) shoot biomass, and (d) root biomass of 6 treatment groups. *t*-test results indicated as *: *p* ≤ 0.05; **: *p* ≤ 0.01; ***: *p* ≤ 0.001.

Box-and-whisker plots coupled with one-way ANOVA followed by *t*-tests were employed to compare the length and biomass of the shoot or root tissues among the 6 treatment groups (Figure 3). The ANOVA tests with p-values smaller than 0.05 for all comparisons (shoot length: *p* = 2.57 × 10^–63^; root length: *p* = 4.10 × 10^–3^; shoot biomass: *p* = 3.47 × 10^–32^; root biomass: *p* = 2.39 × 10^–32^) determined the statistically significant differences between these multiple treatment groups. To identify and understand the magnitude of the observed difference, *t*-tests were performed within the same exposure technique (control vs Cu exposure, control vs Mo exposure, and Cu exposure vs Mo exposure for root exposure and leaf exposure) and between different exposure techniques (root exposure-control vs leaf exposure-control, root exposure-Cu vs leaf exposure-Cu, and root exposure-Mo vs leaf exposure-Mo). Within the root exposure technique, the Mo exposure group was significantly different from the control and Cu exposure groups for all physiology measurements. These statistically significant differences indicated that root exposure to Mo ENMs has a distinct effect on the physiological response. However, exposure to Mo ENMs via the leaves did not have a significant effect compared to the control, indicating that there is a very significant difference depending on the exposure route. The absence of significance might be due to various factors, such as differing absorption rates or sensitivity of tissues to Mo ENMs and Mo ions between roots and leaves.

### 3.2 Metal Accumulation and Distribution

**Figure 4:**
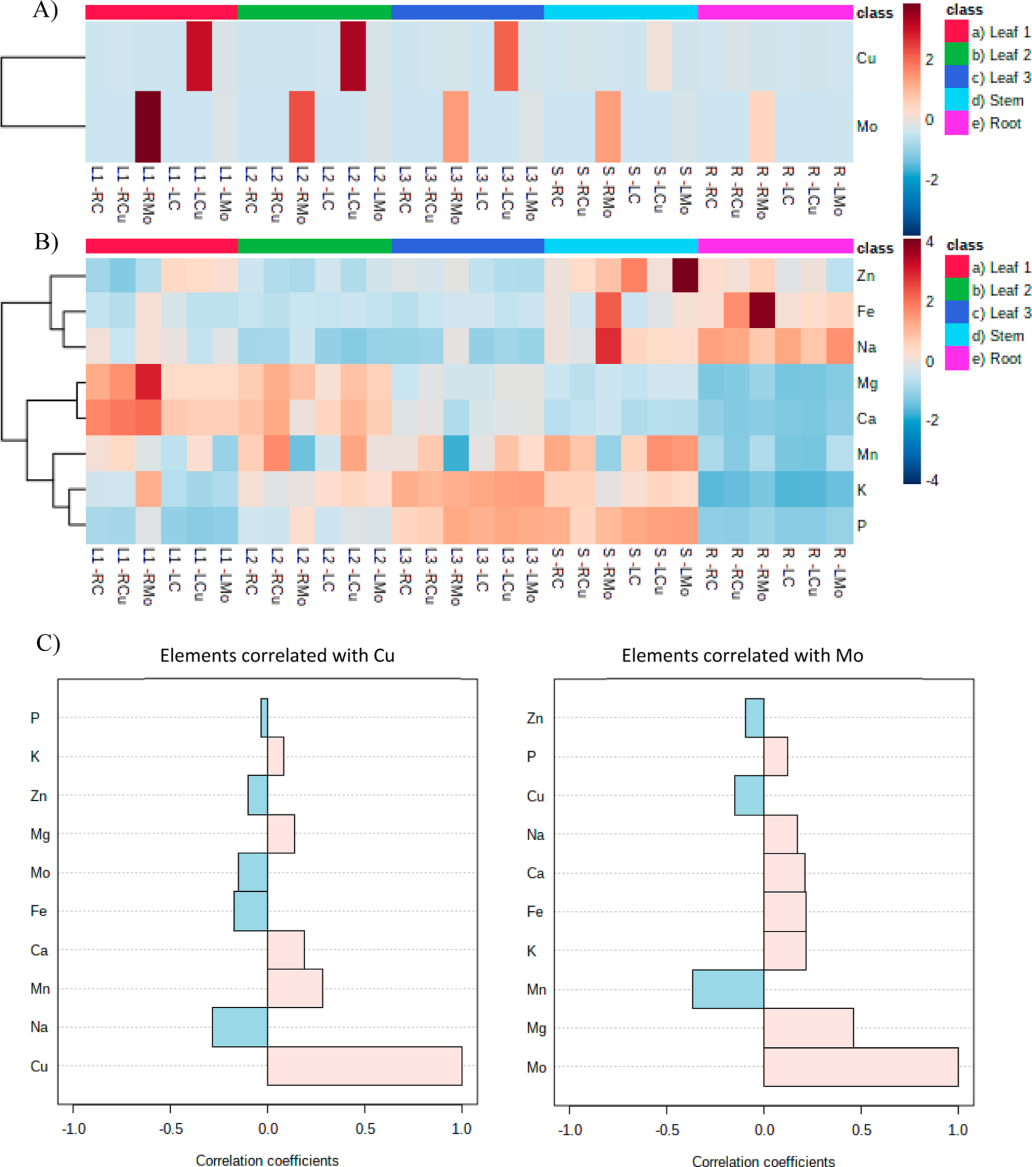
Heatmap of metal concentrations in plant tissues. (A) Cu and Mo concentration in plant tissues; (B) nutrient element concentration in plant tissues. (C) Correlation analysis between Cu and Mo and other nutrient elements. RC: root exposure control; RCu: Cu exposure through root; RMo: Mo exposure through root; LC: leaf exposure control; LCu: Cu exposure through leaf; LMo: Mo exposure through leaf. Element concentration data are listed in Table S3.

The heatmap analysis (Figure 4a,b) presented the concentrations of Cu, Mo, and other nutrient elements in different tissues and exposure scenarios and highlighted some interesting findings regarding the distribution of these elements across different tissues and exposure techniques as well as their potential interactions with other nutrient elements. First, Mo concentration increased significantly with root exposure to Mo-NP, with the highest Mo concentration in leaf 1 (L1) (1823.97 ± 48.45 μg/g), followed by L2 (1178.93 ± 5.05 μg/g), L3 (779.74 ± 2.84 μg/g), stem (S) (757.81 ± 42.84 μg/g), and root (R) (386.53 ± 28.88 μg/g). Leaf exposure to Mo-NP also caused increased Mo concentration (e.g., 89.88 ± 16.05 μg/g in L1), but the effect was less pronounced compared to that in root exposure. It is not surprising since soil application is recommended in agriculture due to the low solubility of molybdenum trioxide (MoO_3_).^32^ The Cu concentration increased significantly with leaf exposure to Cu-NP, with the highest concentration observed in L2 (740.04 ± 23.31 μg/g), followed by L1 (688.92 ± 5.29 μg/g), L3 (493.24 ± 1.77 μg/g), S (89.05 ± 0.42 μg/g), and R (12.24 ± 0.16 μg/g). Meanwhile, the root exposure to Cu-NP only slightly increased the Cu concentration in the root tissues (28.87 ± 0.03 μg/g). These findings illuminate the differential uptake strategies and translocation dynamics of Mo and Cu within the plant. Mo exhibits a strong root-to-leaf translocation, indicating a clear pathway from root uptake to transport in the leaves. Cu, on the other hand, demonstrates a distinct preference for leaf uptake, with less emphasis on accumulation in the roots or translocation. This aligns to a previous study, which observed higher efficiency of Cu uptake through foliar spray rather than via soil irrigation.^33,34^ This distinction highlights the nuanced strategies that plants employ in assimilating different elements.

Moreover, correlation analysis (Figure 4c) reveals different relationships between Cu and Mo and other nutrient elements. For example, there is a strong positive correlation between Mo and Mg concentrations, which suggests that there might be shared uptake or transport mechanisms for these two elements. The strong negative correlation between Mo and Mn concentrations suggests that there might be competitive interaction between these two elements. On the contrary, the weaker correlation between Cu and Mg concentrations, as well as the strong positive correlation between Cu and Mn concentrations, indicates that the relationships between Cu and these elements are distinct from those of Mo. In addition, Na and Fe also show opposite correlations with Mo or Cu concentrations. This suggests that Cu might have different uptake dynamics and interactions compared to those of Mo, which aligns with the observed negative correlation between Mo and Cu (Figure 4C). The antagonistic effects between Cu and Mo uptake have been observed in several plant species, including berseem (Egyptian clover)^35^ and wheat.^36^ The antagonistic effects of Cu with Mo can also explain the leaf yellowing observed in Mo treatment through root (Figure 2C), since the decreased availability of copper due to excess molybdenum uptake could disrupt chlorophyll formation and impair photosynthetic activity owing to the importance of Cu as cofactor of various enzymes in chlorophyll.^37,38^ The correlations observed in our study provide insights into potential elemental interactions and complex nutrient uptake dynamics and transport mechanisms within the wheat plant.

### 3.3 Targeted Proteomics Analysis

**Figure 5:**
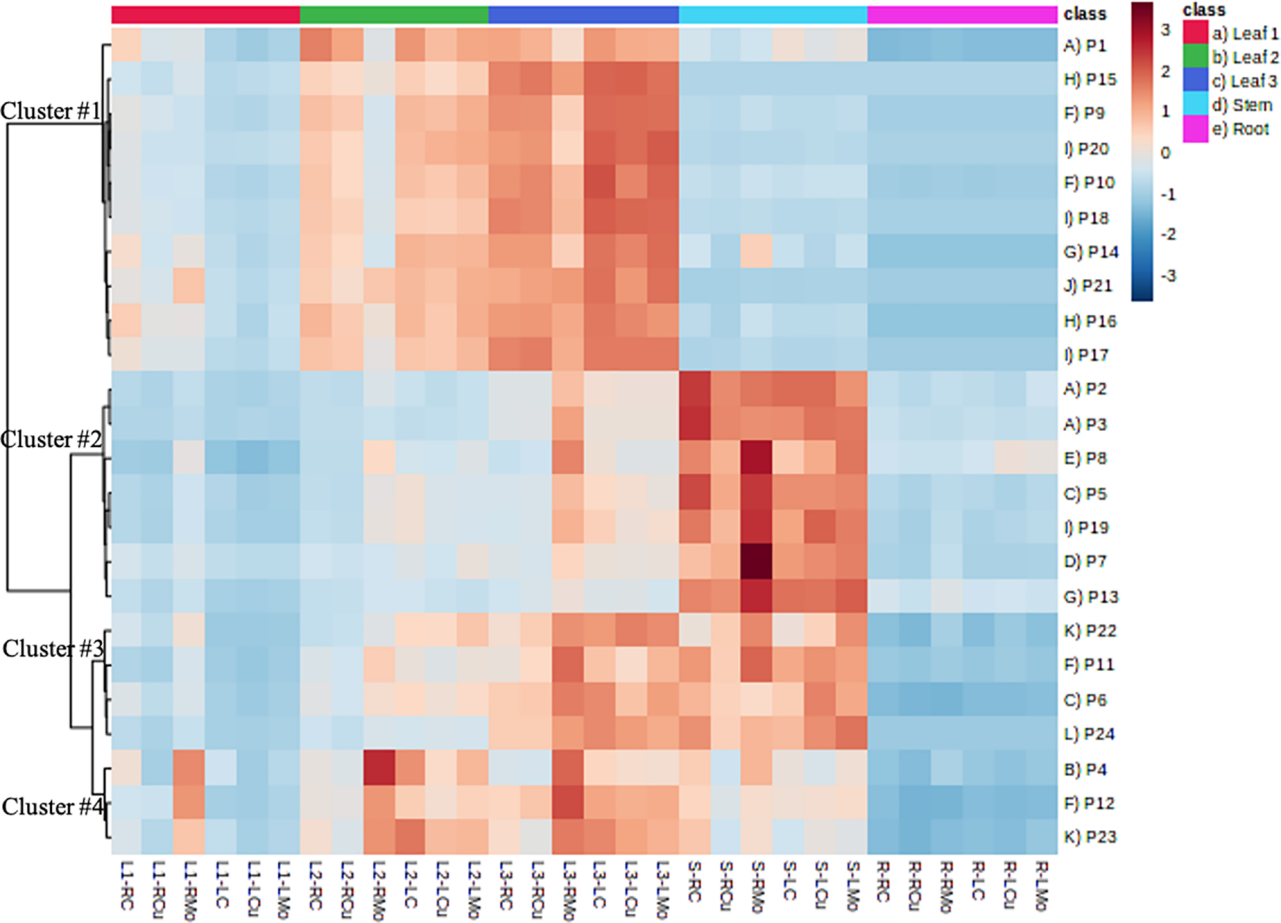
Heatmap of protein concentrations in different plant tissues with different treatments. Refer to Table 1 for the Pathway and Protein IDs.

The heatmap of protein concentrations provided interesting trends of the distribution and clustering patterns of proteins across different tissues and exposure techniques (Figure 5). The first 10 proteins in cluster #1 exhibit a pattern, where L3 has the highest protein concentrations, followed by L2, L1, stem, and roots. Conversely, the 11 proteins in clusters #2 and #3 show a pattern, where stem has the highest concentrations, followed by L3 (very similar as in stem for cluster #3), L2, L1, and roots. The three proteins in cluster #4 have the highest concentrations in L2, closely followed by L3, then S, L1, and roots. Overall, roots and L1 had the lowest protein concentrations, and L3 and S had the highest ones. This suggests tissue-specific distribution patterns for these proteins, indicating that different tissues might have varying protein expression profiles, even among leaves. These observations align with the expected metabolic demands and functional distribution of proteins in different plant tissues. For example, the presence of proteins associated with the Calvin cycle and photosynthesis (e.g., calvin cycle GAP, calvin cycle FBPase, and Calvin cycle aldolase) in cluster #1 is consistent with the higher metabolic activity of these pathways in leaves, which are the most important photosynthetic tissues. In addition, the presence of proteins related to the photorespiratory pathway (e.g., peroxisomal aminotransferases and photosystem II stability/assembly factor HCF136) in cluster #1 further emphasizes the active engagement of leaves in these processes. Moreover, since mitochondrial electron transport and ATP synthesis play a crucial role in synthesizing ATP, which supports the energetic demands of photosynthetic tissues and plant growth,^39^ it is logical to find the related proteins in high concentrations in leaves (e.g., ATP synthase beta subunit and ATP synthase delta chain). However, ATP synthase F1-ATPase (cluster #3) and transport p- and v-ATPase (cluster #4), which are also involved in ATP synthesis pathway, showed high concentration in stems other than leaves. Similar distinction was found for proteins related to amino acid metabolism and N-metabolism, with proteins separately grouped in cluster 1 and cluster #2. This finding suggests that while proteins within the same pathway might have related functions, their expression patterns in different tissues could be influenced by factors beyond their pathway interactions.

**Figure 6:**
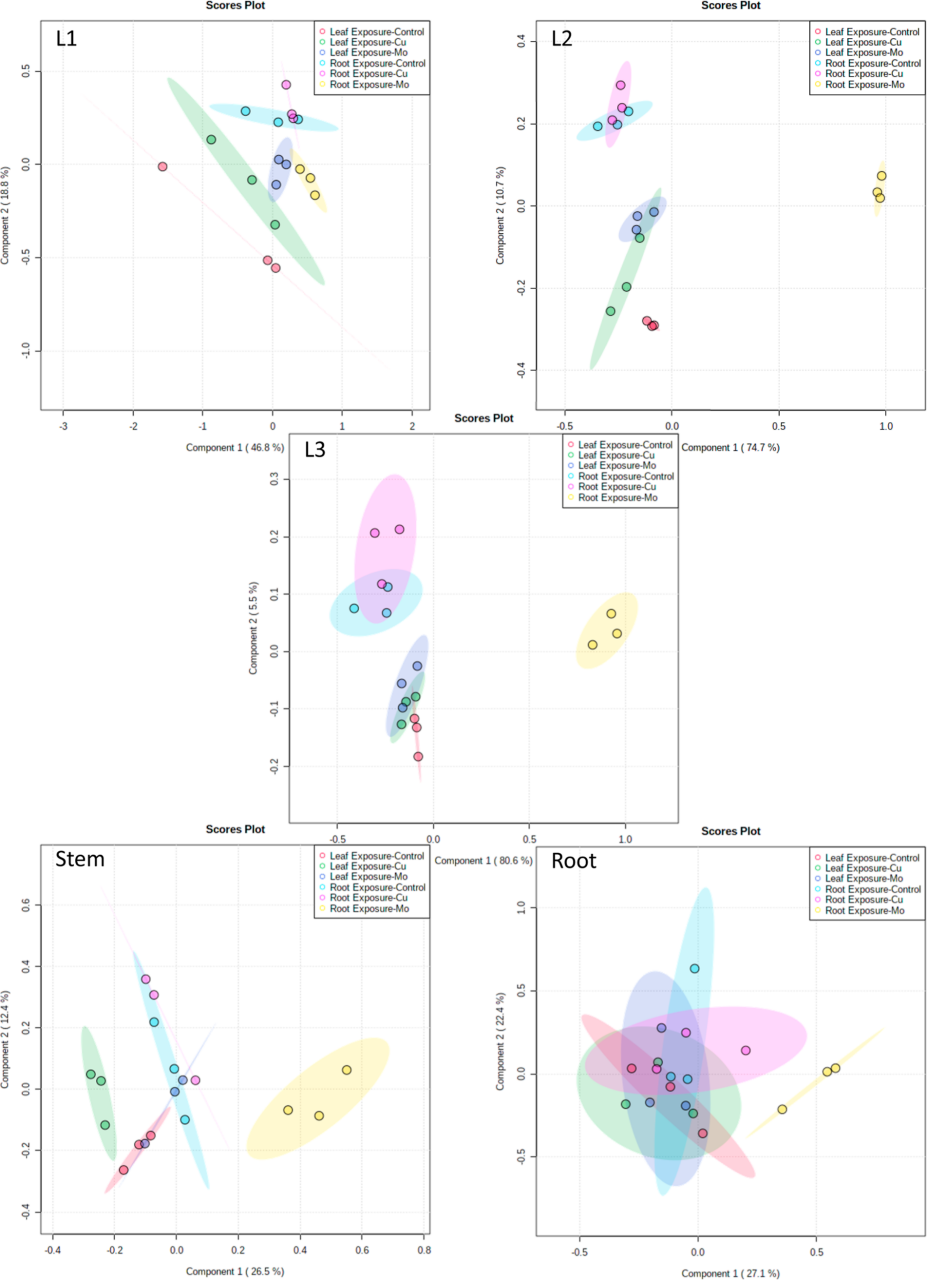
PLS-DA of protein concentrations in each plant tissue with different treatments.

Due to the tissue-specific distribution of proteins, protein concentrations were analyzed within each tissue part. PLS-DA was used to visualize the separation between the six treatment groups at the protein level, which offers an effective means to discern distinct patterns in the proteomic responses (Figure 6). For all tissues, there is a strong separation between root exposure of Mo (yellow dots) from all other treatments. This separation aligns with the pattern observed in physiological measurements, reinforcing the idea that Mo exposure through root has a distinct impact on plant response across different levels of analysis. In addition, it shows separation between treatment groups based on exposure techniques (e.g., red vs blue), which suggests that the choice of exposure method (leaf exposure vs root exposure) has a discernible effect on the proteomic responses of the plants. This separation also aligns with physiological measurements and metal analysis, and it supports the notion that the exposure approach itself influences the proteomic profiles, indicating that different tissues and pathways might be engaged based on how the exposure occurs.

**Figure 7:**
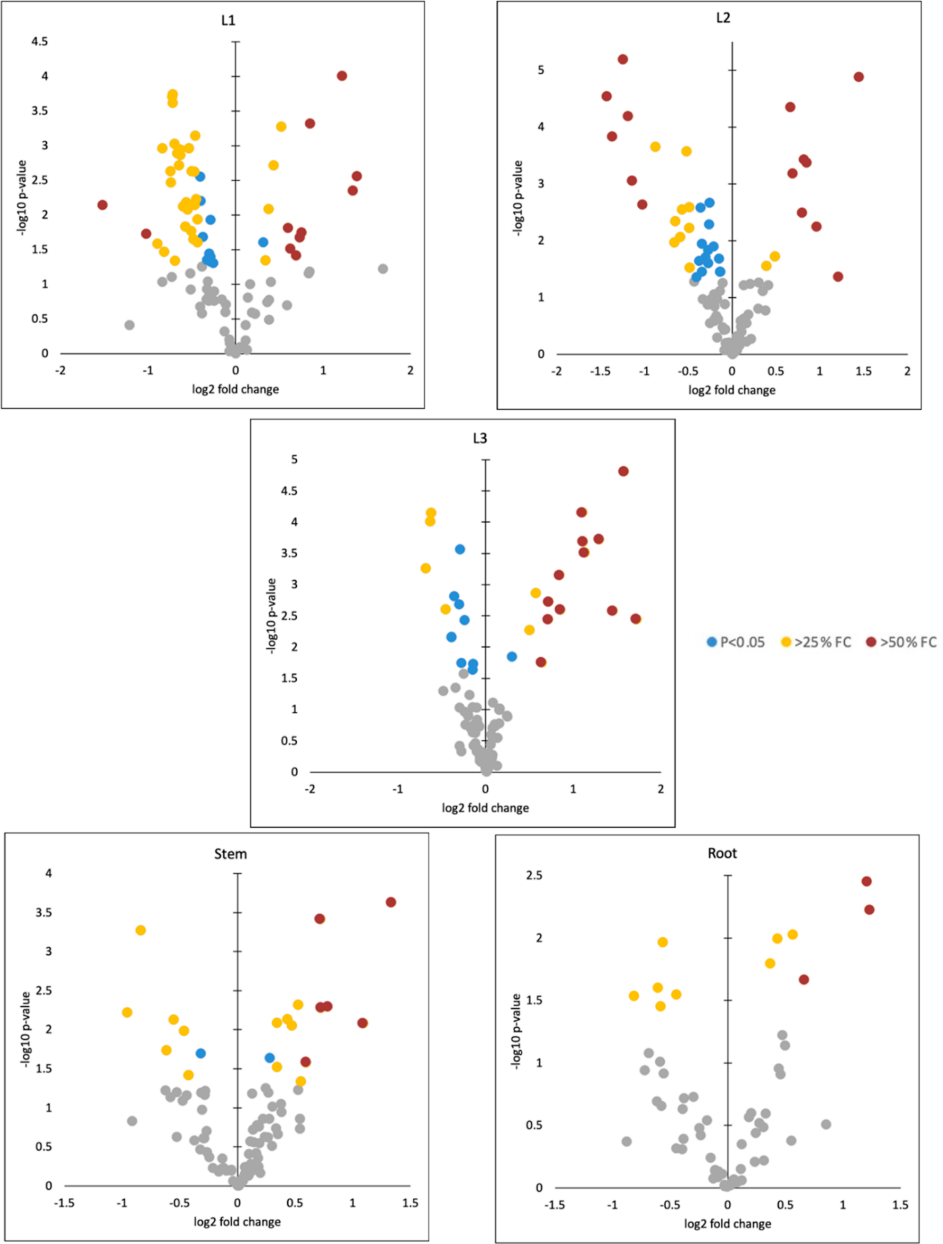
Volcano plots to visualize the relationship between significance (*p*-values <0.05) and FCs in each tissue. Gray points: not significant; blue color points: significant but 0.75 < FC < 1.25; yellow color points: significant and FC ≥ 1.25 or ≤0.75; red color points: FC ≥ 1.5 or ≤0.5.

To quickly identify the proteins that exhibit both substantial changes in expression and statistical significance, volcano plots were used to visualize the relationship between significance (*p*-values) and FCs in each tissue (Figure 7). Gray spots represent data points with *p*-values greater than 0.05, indicating that these changes are not statistically significant. Blue points indicate significant changes with 0.75 < FC < 1.25. While these changes are statistically significant, their relatively small magnitude suggests that they might not have a substantial impact on the biological response. Yellow and red points represent significant changes with FC ≥ 1.25 or ≤0.75 (yellow) and FC ≥ 1.5 or ≤0.5 (red), which represent alterations in protein expression that are both statistically significant and biologically relevant due to their considerable magnitude.

**Figure 8:**
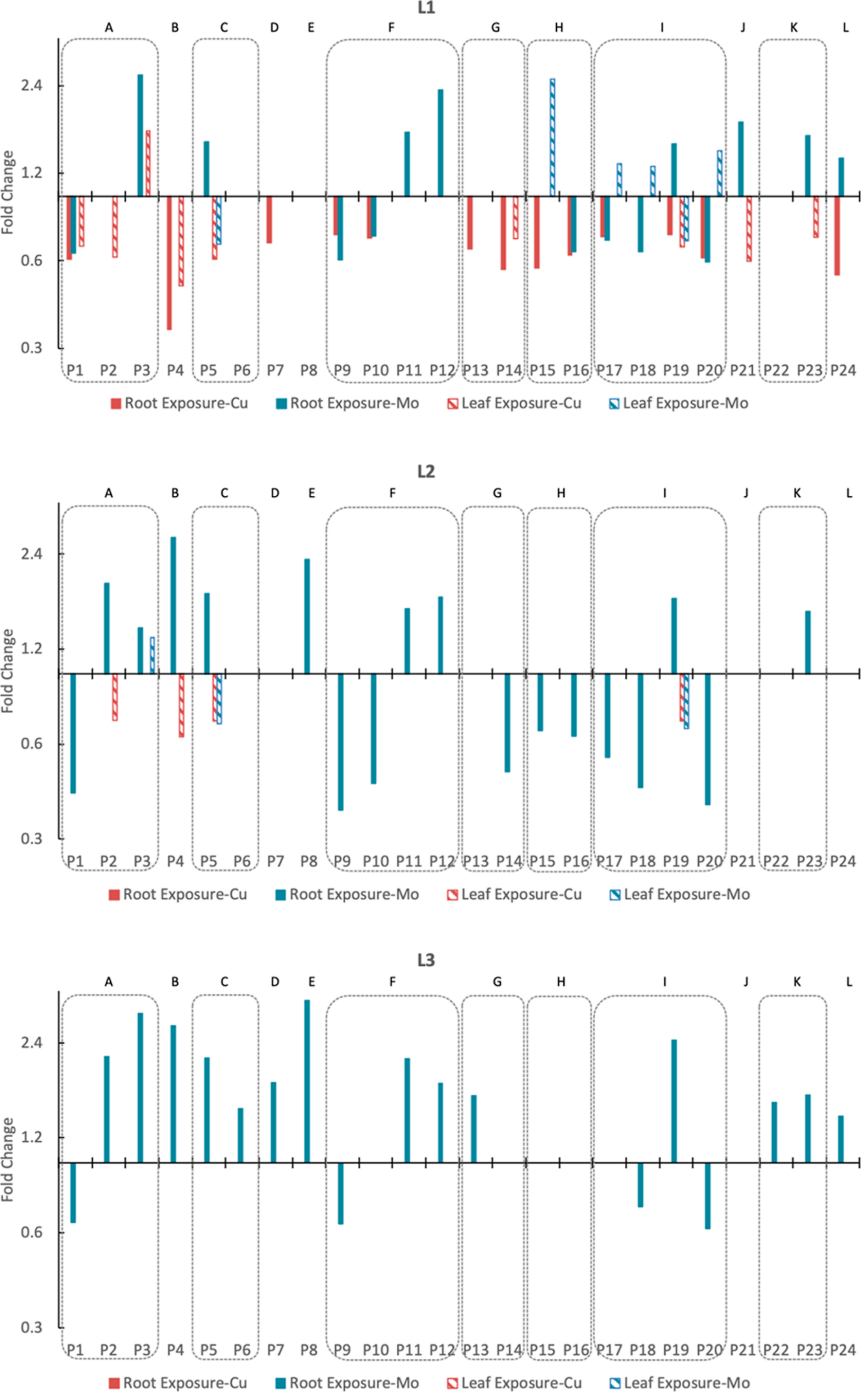
FC bar plots of proteins with FC ≥ 1.25 or ≤0.75 significant changes in different plant tissues.

To better interpret the results, the data was filtered, and FC bar plots were made focusing on the yellow and red data points (Figure 8). Proteins exhibited different regulation patterns between Cu and Mo exposures, particularly in leaf tissues (L1–L3), where Mo causes protein upregulation and Cu causes downregulation. This finding highlights the specificity of protein responses to different metal exposures. Moreover, most of the regulation caused by Mo is through root exposure, which aligns with the elemental concentration and emphasizes the significance of root exposure of Mo in driving protein expression changes. In addition, the pattern of regulation activity from high to low in leaves to roots aligns with the metal release from the Mo ENMs, Mo uptake, and translocation results as well. This suggests that the physiological and molecular responses of different tissues are connected, with leaves being the most sensitive and responsive, possibly due to their prominent role in Mo accumulation. Another interesting finding is that proteins within the same metabolic pathway can have diverse regulation patterns (e.g., within pathway F, P9 and P10 downregulated, while P11 and P12 upregulated). This suggests that even within the same metabolic pathway, the expression levels of individual proteins can be regulated independently.

**Figure 9:**
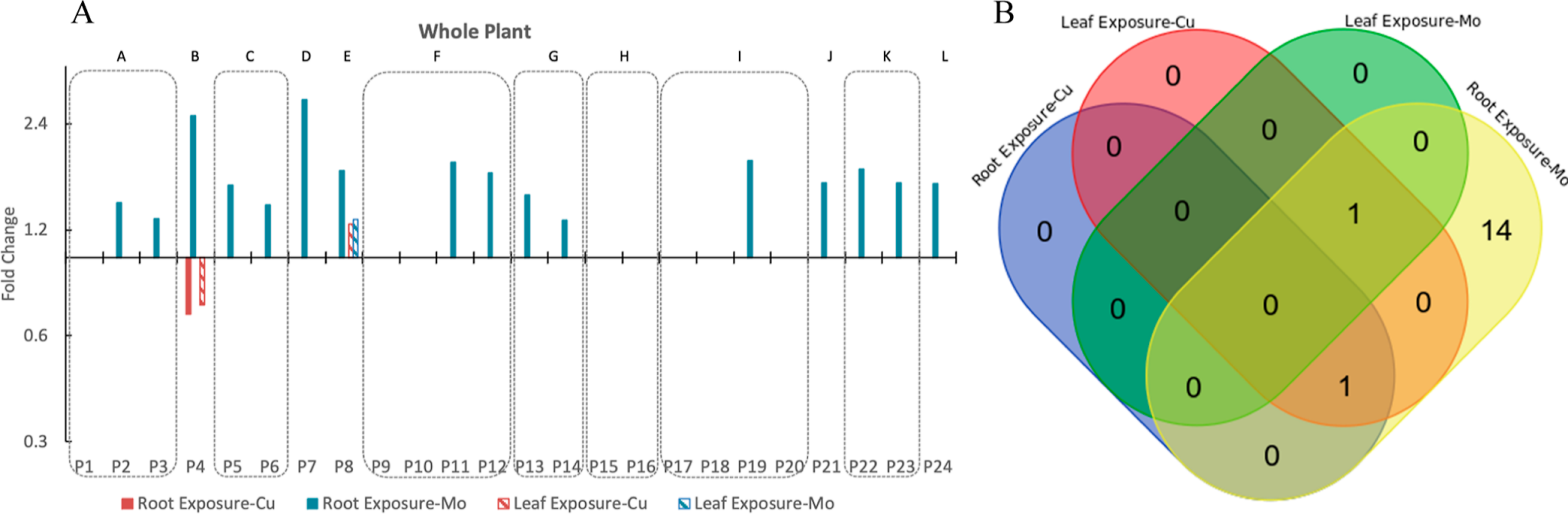
Protein expression in the whole plant. (A) FC bar plot of proteins with FC ≥ 1.25 or ≤0.75 significant changes in the whole plant; (B) Venn diagram of proteins with FC ≥ 1.25 or ≤0.75 significant changes in the whole plant.

To get a comprehensive overview of the changes occurring in the entire plant in response to ENM exposure, targeted protein concentrations for the whole plant were calculated by adjustment of the biomass distribution (Figure 2B) for five different tissues. It shows that among 24 selected proteins (involved in 12 metabolic pathways), 16 proteins (involved in 11 metabolic pathways) were significantly upregulated (1.28 < FC < 2.81) under Mo ENM exposure through the roots (Figure 9). This is not surprising as Mo is an essential trace element necessary for various plant metabolic processes, and it is a key component of enzymes involved in nitrogen fixation, nitrate reduction, and amino acid metabolism, which are all fundamental processes that support plant growth and development.^40,41^ Increased Mo concentrations can potentially lead to higher activity rates of reactions of Mo-dependent enzymes, which play crucial roles in metabolic pathways. This, in turn, could upregulate the proteins involved in the metabolic pathways in plants. The coordinated upregulation of multiple pathways suggests the presence of a complex regulatory network that senses Mo availability and coordinates responses across various pathways to ensure optimal metabolic function. However, the excessive presence of Mo in the soil, which is then translocated to the leaves in excess, leads to a negative physiological response (yellowing and stunted growth). The upregulation of proteins could be a strategy to increase Mo tolerance in wheat plants. For example, transport H+ transporting pyrophosphatase (P7), which was the most upregulated protein for the entire plant, has also been reported to upregulate to enhance proton pump expression, improving tolerance to the toxicity of cadmium in tobacco plants.^42^

In contrast to Mo exposure, Cu exposure has a relatively smaller impact on protein expression at the whole plant scale since only two proteins showed significant changes (Figure 9). However, 18 proteins (involved in 11 pathways) showed significant changes particularly in leaf tissues and when exposure was via leaves (Figure 8), which indicates that Cu has a more localized impact particularly. This result correlated well with the high copper concentrations in leaves exposed via this route, which can lead to oxidative stress in plant cells due to the generation of reactive oxygen species (ROS).^43^ The mostly downregulated proteins (root exposure: 0.35 < FC < 0.74; leaf exposure: 0.49 < FC < 0.72) observed in response to Cu exposure in leaves suggests that the plant initiated specific responses to mitigate the effects of copper-induced oxidative stress. This includes regulating the expression of enzymes like catalase (P21), a vital enzyme in the cellular defense against oxidative stress by efficiently breaking down hydrogen peroxide, to help protect cells from the damaging effects of ROS, contributing to overall cellular health and function.^44,45^

### 3.4 Effect of Exposure to High vs Low Mo ENM Concentrations through Root

The significant upregulation of most selected proteins under Mo exposure through root indicates that the plant is actively responding to the presence of molybdenum. However, the depressed physiological measurements, despite protein upregulation, suggest that excess molybdenum negatively affected plant health. Yellowing of leaves and depressed root growth were also reported in a hydroponic experiment investigating the uptake of Mo in cress (*Lepidium sativum* L.) with 7000 μg/L Mo exposure, which was 35 times higher than the optimal dose.^46^ To further study the dose-dependent response of root exposure to Mo ENMs, a lower concentration of 0.6 mg of Mo/plant was added to the experiment. The physiological measurements of the low Mo exposure group, including total biomass and biomass distribution (Figure S1A), leaf color (Figure S1B), and shoot and root length (Figure S1C), were not significantly different from those of the control but were significantly different from those of the high Mo exposure group. The slight and significant increase in shoot biomass and the decreased root biomass under low Mo exposure suggest that a lower concentration of Mo improves plant health compared to the control, and the difference between the low and high Mo exposure groups is substantial. In terms of metal uptake, plants with low Mo exposure exhibit even higher Mo concentrations in leaves (1.77, 2.67, and 3.40 times higher in L1, L2, and L3, respectively) than those with high exposure (Figure S2). This surprising observation implies that nutrient uptake by plants follows complex kinetics, and at lower Mo concentrations, plants might enhance their uptake mechanisms.

At the protein level, there is a similar tissue-specific distribution of proteins (Figure S3) as noticed in Figure 5, which suggests that the distribution pattern was determined by the metabolic demands and functions in each tissue. However, the clear separations observed in the PLS-DA between dose-specific treatments underscore the distinct molecular responses triggered by the different Mo concentrations (Figure S4). Particularly, in contrast to the upregulation with high Mo exposure, the proteins were significantly downregulated under low Mo exposure, especially in L1 (0.23 < FC < 0.68) and stem (0.13 < FC < 0.68) tissues (Figure S5). Considering the protein expression in the whole plant using the adjustment of biomass distribution, levels of 5 (P7, P8, P14, P21, and P22) out of the 16 significantly regulated proteins were consistently upregulated in response to both high and low Mo exposure (Figure S6). Specifically, the involvement of proteins in pathways like N-metabolism (P14), redox (P21), and TCA cycle (P22) processes underscores their pivotal role in harnessing the growth-promoting benefits of Mo. However, proteins P13 and *P*23, also involved in the N-metabolism and the TCA cycle, displayed a contrasting response: downregulated with low Mo exposure but upregulated with high Mo exposure. In addition, proteins P9, P10, and P20, crucial for processes like mitochondrial electron transport, ATP synthesis, and the Calvin Cycle, demonstrated no significant change in levels under high Mo exposure but were downregulated under low Mo exposure. These findings indicate a complex relationship between Mo availability and these metabolic processes and a potential requirement for higher Mo levels to effectively drive these energy-related pathways. The opposite trends in protein regulation indicate that the plant is employing distinct strategies to adapt to varying Mo levels, such as optimizing nutrient uptake, altering metabolic pathways, and fine-tuning stress responses. The dose-specific regulation was also reported in a previous study on metabolomic responses of corn and wheat plants due to exposure to 8 or 40 mg Mo/plant.^28^ This investigation of the dose effects underscores the fine balance between nutrient stimulation and toxicity. While a high dose of Mo induced significant protein upregulation, it also yielded depressed physiological measurements, highlighting the importance of appropriate nutrient dosing for optimal plant health.

In conclusion, this study delved into the response of wheat plants to a Mo-based nanofertilizer and a Cu-based nanopesticide through a comprehensive exploration of various aspects, including physiological measurements, metal uptake and translocation, and protein expression. Exposure to Mo ENMs, which release substantial amount of Mo ions, results in significant Mo root uptake and translocation to leaves, which results in significant upregulation of multiple proteins involved in diverse metabolic pathways, particularly those related to photosynthesis and the Calvin cycle, ATP synthesis, N-metabolism, redox, and TCA cycle. This aligns with the pivotal role of Mo as a cofactor for enzymes essential in nitrogen fixation, amino acid biosynthesis, and other fundamental plant processes. Notably, the study highlighted a dose-dependent response, where a higher dose of Mo through root exposure induced robust upregulation of proteins, albeit yellowing and stunted growth, while a lower dose resulted in more translocation but surprisingly induced downregulation of some proteins. The low Mo exposure induced downregulation of these proteins, mostly involved in energy metabolism and carbon fixation, suggesting the requirement of higher levels of Mo to maintain their activity effectively. In contrast, Cu ENM exposure demonstrated a distinct pattern. While fewer proteins exhibited significant changes at the whole plant level, the study unveiled pronounced effects on leaf tissues, notably from exposure via leaves. This underlines that while Cu ENMs provide the plant protection in terms of fungi and other pests, Cu ENMs have the potential to initiate stress responses and metabolic adaptations, particularly in the initial stages of exposure. To delineate and validate the mechanistic differences arising from the nanostructures, future studies incorporating non-nanoscale Cu and Mo controls alongside nanoscale exposures can help better elucidate nanospecific effects.

This study leveraged targeted proteomics to gain highly quantitative insight into the nuanced response of plants to Cu and Mo ENM exposure. The analysis at the tissue level provided a more granular understanding of these responses, allowing us to discern tissue-specific variations that would have been overlooked in a whole-plant approach. This precision was invaluable in unraveling the intricate metabolic shifts triggered by the Cu and Mo availability. Furthermore, the integration of metabolomics, which delved into the uptake and translocation of Cu and Mo, enriched our understanding by providing a comprehensive view of nutrient dynamics within the plant. The study’s contribution involves not only unraveling the proteomic response of wheat under Mo and Cu ENM exposure but also illuminating the potential applications and risks associated with their utilization in agriculture. The findings hold relevance for optimizing nutrient supplementation strategies to enhance crop productivity while minimizing adverse effects on plant growth.

## Supporting Information Available

Detailed information on the HPLC and MS conditions, transitions and LOD for each peptide for targeted proteomics analysis with LC–MS/MS, data of metal concentration in different plant tissue samples with different treatments, physiology measurements, metal uptake, and translocation, and proteomics of plants exposed to high and low Mo ENM treatments (PDF)
