# Supplementary material for: Assessing the Impacts of Cu and Mo Engineered Nanomaterials on Crop Plant Growth Using a Targeted Proteomics Approach
Source: ACS Agric Sci Technol. 2023 Dec 22;4(1):103–17. doi: 10.1021/acsagscitech.3c00431 (PMC10792604; doi:10.1021/acsagscitech.3c00431)
Supplement: Supplementary file 1 — as3c00431_si_001.pdf [file as3c00431_si_001.pdf]

Supporting Information for

Assessing the Impacts of Cu and Mo Engineered Nanomaterials on Crop Plant Growth Using a Targeted Proteomics Approach

Weiwei Li and Arturo A. Keller*

Bren School of Environmental Science and Management, University of California at Santa Barbara, Santa Barbara, California 93106, USA

*Corresponding author: Tel: +1 805 893 7548; Fax: +1 805 893 7612. Email address: arturokeller@ucsb.edu

Table S1. HPLC conditions and MS conditions for LC-MS/MS analysis method.

**HPLC Conditions**		
Column	Agilent Polaris 3 C18-Ether 150x3.0mm (p/n:A2021150X030)	
Mobile phase A	Water + 0.1% (v:v) formic acid +3% (v:v) DMSO	
Mobile phase B	Acetonitrile + 0.1% (v:v) formic acid +3% (v:v) DMSO	
Flow rate	0.40 mL/min	
Column temperature	25 °C	
Injection volume	2 μL	
Total run time	14 minutes	
Gradient	Time (min)	%B
	0.00	5
	10.00	70
	10.01	5
	14.00	5
**MS Conditions**		
Ionization mode	ESI Positive	
Gas temperature	340 °C	
Gas flow	12 L/min	
Nebulizer	40 psi	
Sheath gas temperature	250 °C	
Sheath gas flow	9 L/min	
Capillary voltage	Positive	Negative
	3,500 V	3,500 V
Nozzle voltage	Positive	Negative
	2,000 V	2,000 V

Table S2. Transitions, LOD and MDL for each peptide.

ID	Sequence	Retention	Precursor ion (m/z)	Product ions					LOD (ng/mL)	MDL (ng/g)
				Quant	Collision	Qual	Collision	Fragm		
	**Peptides**									
1	IQNGGTEVVEAK	6.42	623.2	242.1	20	86.1	32	132	0.02	0.08
2	SVHEPMQTGLK	6.70	409.8	110.2	40	84.0	40	96	0.41	2.05
3	TAVAAVPYGGAK	7.00	553.1	173.0	24	72.1	40	112	0.08	0.40
4	LVGVSEETTTGVK	7.12	660.7	86.0	36	72.1	40	137	0.09	0.44
5	VAEGDAEDVDRAVVAAR	7.19	582.0	786.9	16	72.0	40	96	0.16	0.81
6	KALDYEELNENVK	7.25	522.6	102.0	16	86.2	16	96	0.24	1.18
7	SGDVYIPR	7.31	454.0	548.3	16	60.1	40	96	0.01	0.04
8	GMAVPDSSSPYGVR	7.42	712.3	260.0	28	189.2	36	132	0.02	0.09
9	GNATVPAMEMTK	7.43	625.7	172.0	40	70.0	40	117	0.10	0.51
10	EFAPSIPEK	7.46	509.6	335.7	16	70.0	40	96	0.10	0.49
11	FASINVENVEDNRR	7.51	555.3	120.0	24	191.0	16	96	0.00	0.02
12	FVIGGPHGDAGLTGR	7.60	485.5	604.4	12	120.0	32	96	0.00	0.01
13	AADNIPGNLYSVK	7.79	681.8	877.4	20	230.0	32	127	0.08	0.38
14	TVVSIPNGPSELAVK	8.05	756.4	172.8	40	200.9	36	132	0.01	0.06
15	TLGELPAGSVIGSASLRR	8.12	595.7	635.9	16	186.9	20	117	0.00	0.02
16	VAEFSFR	8.13	428.5	171.0	12	72.1	24	96	0.01	0.03
17	YIGSLVGDFHR	8.13	422.1	494.3	8	86.0	28	96	0.01	0.03
18	TALIDEIAK	8.21	487.5	173.0	12	86.0	40	112	0.01	0.03
19	VAPEVIAEYTVR	8.21	674.3	589.0	16	70.0	40	147	0.06	0.28
20	AAVIGDTIGDPLK	8.23	635.7	72.0	32	86.0	32	132	0.06	0.30
21	IGGLTLNELGR	8.40	572.2	228.0	24	86.1	40	122	0.01	0.07
22	TLAEEVNQAFR	8.45	639.7	187.1	28	215.0	20	127	0.04	0.21
23	IGLFGGAGVGK	8.59	488.5	545.2	16	86.1	24	117	0.01	0.05
24	VQLLEIAQVPDEHVNEFK	8.62	703.8	227.8	24	72.1	36	142	0.01	0.06
25	TAIAIDTILNQK	8.68	651.3	173.1	24	86.0	40	112	0.10	0.50
26	KPWNLSFSFGR	8.79	670.3	84.0	36	70.1	40	137	1.17	5.84
27	TWPEDVVPLQPVGR	8.96	797.4	653.7	20	342.1	40	147	1.55	7.75
28	ADGGLWLLVR	9.63	550.7	159.0	40	86.0	40	117	0.02	0.11
	**Internal Standards**									
2*	SVHEPMQTGLK{Lys(13C6,15N2)}	6.69	412.5	90.1	40	69.9	40	96		
7*	SGDVYIPR{Arg(13C6,15N4)}	7.31	459.0	558.3	12	260.0	16	91		
18*	TALIDEIAK{Lys(13C6,15N2)}	8.21	491.6	172.8	16	86.0	40	81		
26*	KPWNLSFSFGR{Arg(13C6,15N4)}	8.79	675.3	84.1	36	70.0	40	137		

Table S3. Metal concentration in different plant tissue samples with different treatments.

Sample ID	Exposure Elements (μg/g)		Nutrient Elements (μg/g)							
	Cu	Mo	K	Mg	Ca	P	Mn	Fe	Zn	Na
L1-RC	2.05± 0.00	0.75± 0.02	5554.72± 221.67	1155.45± 4.30	556.85± 8.92	170.68± 7.36	34.27± 0.02	11.99± 0.15	2.77± 0.02	41.63± 0.35
L1-RCu	5.17± 0.04	0.76± 0.03	5516.42± 83.19	1304.96± 50.74	585.87± 4.57	155.52± 7.03	37.55± 1.53	9.05± 0.12	1.83± 0.11	29.36± 1.58
L1-RMo	3.43± 0.77	1822.97± 48.45	9151.55± 180.25	1808.21± 17.94	605.34± 4.80	369.21± 10.54	30.75± 0.65	26.15± 0.23	3.41± 0.00	43.19± 0.34
L1-LC	1.92± 0.15	1.03± 0.04	4815.98± 20.84	849.81± 18.03	397.01± 0.57	91.97± 0.74	27.24± 0.31	17.35± 0.01	6.85± 0.18	38.75± 1.20
L1-LCu	688.92± 5.29	1.59± 0.03	4256.47± 3.47	848.10± 5.76	385.13± 3.48	67.25± 3.10	35.19± 0.14	10.57± 0.03	6.62± 0.12	27.89± 0.01
L1-LMo	28.08± 1.97	89.88± 16.05	4630.07± 374.73	859.26± 146.26	385.17± 46.06	84.64± 34.55	22.94± 4.15	10.64± 1.27	6.07± 1.26	36.14± 6.61
L2-RC	1.83± 0.00	0.65± 0.11	6713.77± 629.39	947.88± 179.00	413.88± 56.11	307.51± 79.21	39.39± 7.65	12.86± 1.67	4.15± 1.57	20.17± 4.15
L2-RCu	2.68± 0.04	1.28± 0.00	6002.09± 3.41	1143.59± 1.56	479.77± 1.57	296.05± 0.98	49.67± 0.07	10.25± 0.01	3.30± 0.36	21.29± 0.27
L2-RMo	1.68± 0.01	1178.93± 5.05	6354.39± 76.71	1014.21± 9.82	282.08± 0.74	502.39± 3.22	19.10± 0.20	13.59± 0.10	3.18± 0.06	30.98± 0.65
L2-LC	6.75± 0.99	0.48± 0.02	6969.96± 647.16	765.70± 80.46	363.13± 52.05	287.49± 43.17	29.67± 3.04	12.70± 1.77	4.34± 0.75	16.06± 2.16
L2-LCu	740.04± 23.31	1.61± 0.05	7578.29± 536.84	1024.16± 32.83	447.87± 34.68	362.75± 16.56	45.70± 1.82	14.06± 1.10	3.28± 0.33	13.59± 0.19
L2-LMo	26.05± 2.20	85.11± 16.98	6985.79± 505.81	927.34± 187.37	389.66± 47.04	351.66± 105.24	33.09± 6.45	12.41± 1.41	4.44± 1.49	16.75± 3.42
L3-RC	3.72± 0.00	0.50± 0.05	9218.28± 645.71	528.30± 39.64	251.10± 24.98	581.94± 47.89	33.48± 2.51	9.84± 0.98	4.86± 0.85	17.54± 1.73
L3-RCu	4.31± 2.24	0.54± 0.09	8749.80± 617.85	644.38± 120.82	269.03± 29.49	621.63± 129.43	40.21± 7.62	13.27± 1.48	4.55± 1.42	18.81± 3.58
L3-RMo	18.56± 0.14	779.74± 2.84	9120.38± 6.14	572.64± 5.19	165.84± 4.54	809.35± 4.58	15.66± 0.00	15.38± 0.10	5.07± 0.11	38.02± 0.50
L3-LC	2.80± 1.01	0.59± 0.02	9456.88± 100.81	570.60± 46.79	238.61± 9.08	778.90± 67.19	31.94± 2.59	15.07± 0.44	3.82± 0.73	16.48± 1.30
L3-LCu	493.24± 1.77	1.97± 0.01	9646.79± 64.27	660.81± 7.25	262.01± 1.02	833.01± 7.89	41.28± 0.24	11.91± 0.19	3.38± 0.03	19.74± 0.16
L3-LMo	20.12± 0.10	98.75± 0.52	9764.08± 2.38	653.44± 0.51	263.13± 7.49	806.52± 13.66	36.53± 0.30	13.85± 0.28	3.25± 0.09	17.66± 0.41
S-RC	4.09± 2.33	0.60± 0.12	7746.65± 529.05	528.66± 93.30	180.92± 22.15	783.85± 155.13	45.67± 8.59	23.35± 2.83	5.56± 1.46	40.67± 7.51
S-RCu	19.20± 0.26	0.48± 0.06	7772.32± 1925.10	466.56± 46.16	200.30± 59.21	580.22± 74.76	40.73± 3.99	15.48± 4.49	6.59± 0.71	35.78± 4.54
S-RMo	5.26± 2.12	757.81± 142.84	6149.11± 543.98	552.50± 112.10	220.60± 22.53	728.94± 159.88	22.79± 4.34	69.77± 8.06	7.91± 0.66	101.68± 21.50
S-LC	2.41± 0.02	0.57± 0.03	6870.02± 5.98	500.54± 0.94	166.05± 2.89	810.60± 14.99	38.49± 0.41	14.02± 0.07	10.75± 0.23	51.14± 0.17
S-LCu	89.05± 0.42	1.50± 0.02	7574.34± 62.51	560.95± 1.45	180.64± 4.09	854.80± 6.39	49.09± 0.34	19.00± 0.17	5.71± 0.03	49.06± 0.09
S-LMo	13.01± 0.01	70.63± 0.22	7308.35± 404.93	565.45± 4.81	176.71± 12.84	864.13± 8.37	47.61± 0.17	26.19± 1.88	17.22± 0.11	45.78± 0.11
R-RC	9.48± 0.19	0.17± 0.00	2662.01± 261.67	221.47± 7.75	116.07± 13.89	88.30± 5.00	25.28± 0.97	27.34± 2.59	6.33± 0.00	70.15± 1.17
R-RCu	28.87± 0.03	0.14± 0.00	2872.15± 5.01	242.19± 2.28	91.84± 1.97	78.51± 1.92	21.03± 0.19	56.87± 0.25	5.81± 0.28	67.67± 0.36
R-RMo	10.13± 0.06	386.53± 28.88	3250.91± 81.64	335.95± 25.04	104.00± 7.33	129.75± 18.26	25.60± 1.60	101.91± 5.84	7.15± 0.08	55.92± 3.25
R-LC	8.63± 0.13	0.16± 0.02	2554.23± 3.43	203.50± 4.81	90.18± 2.14	62.55± 3.93	19.93± 0.51	26.29± 0.17	4.98± 0.09	68.83± 1.14
R-LCu	12.24± 0.16	0.18± 0.01	2619.34± 22.56	208.30± 1.08	84.19± 5.80	63.14± 2.44	19.74± 0.18	29.51± 0.48	5.55± 0.01	57.31± 0.09
R-LMo	8.21± 1.51	13.11± 1.85	2944.04± 141.16	261.63± 38.92	108.47± 3.02	105.13± 28.60	26.57± 3.85	33.55± 1.80	3.70± 1.67	75.01± 8.57

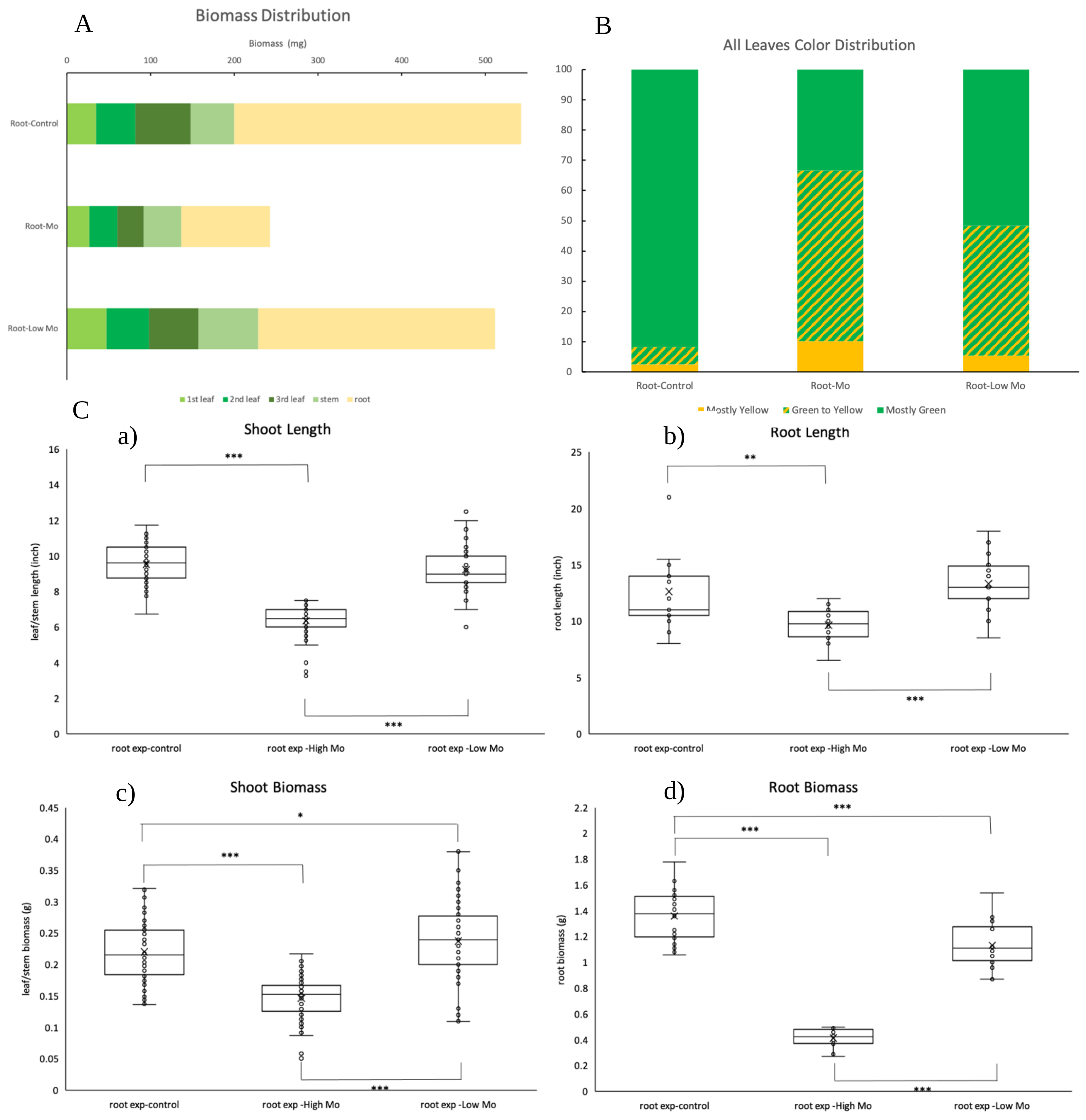

Figure S1. Physiology measurements of plants with high and low Mo exposure treatments. A) Biomass distribution; B) Leaves color distribution; C) The box-and-whisker plot of a) shoot length, b) root length, c) shoot biomass and d) root biomass of Mo treatment groups through root exposure (Control, High Mo and Low Mo). T-test results indicated as *: $p \leq 0.05$; **: $p \leq 0.01$; ***: $p \leq 0.001$.

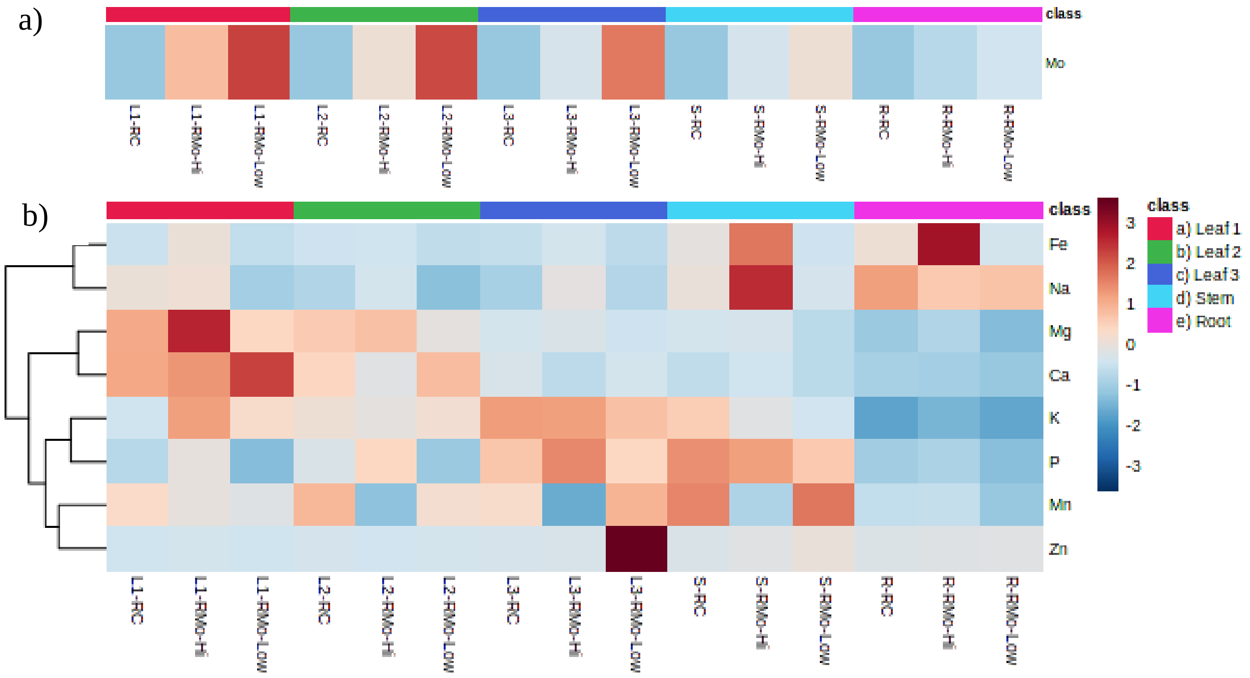

Figure S2. Heatmap of metal concentration in plant tissue samples with high and low Mo exposure treatments. a) Mo concentration in plant tissues; b) Nutrient elements concentration in plant tissues. RC: root exposure control; RMo-Hi: root exposure to Mo-NP with high dose; RMo-Low: root exposure to Mo-NP with low dose.

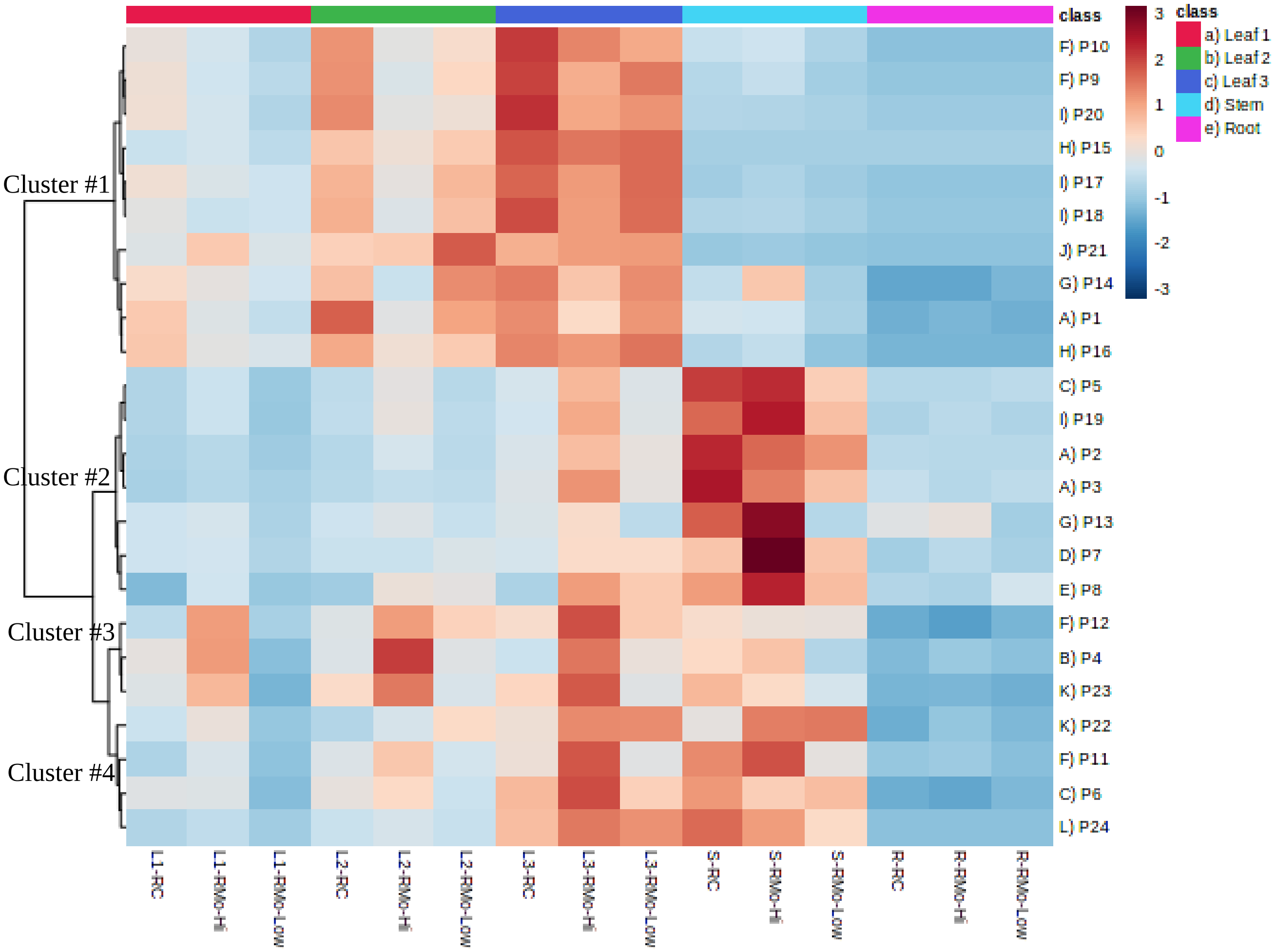

Figure S3. Heatmap of protein concentrations in plant tissues with high and low Mo exposure treatments.

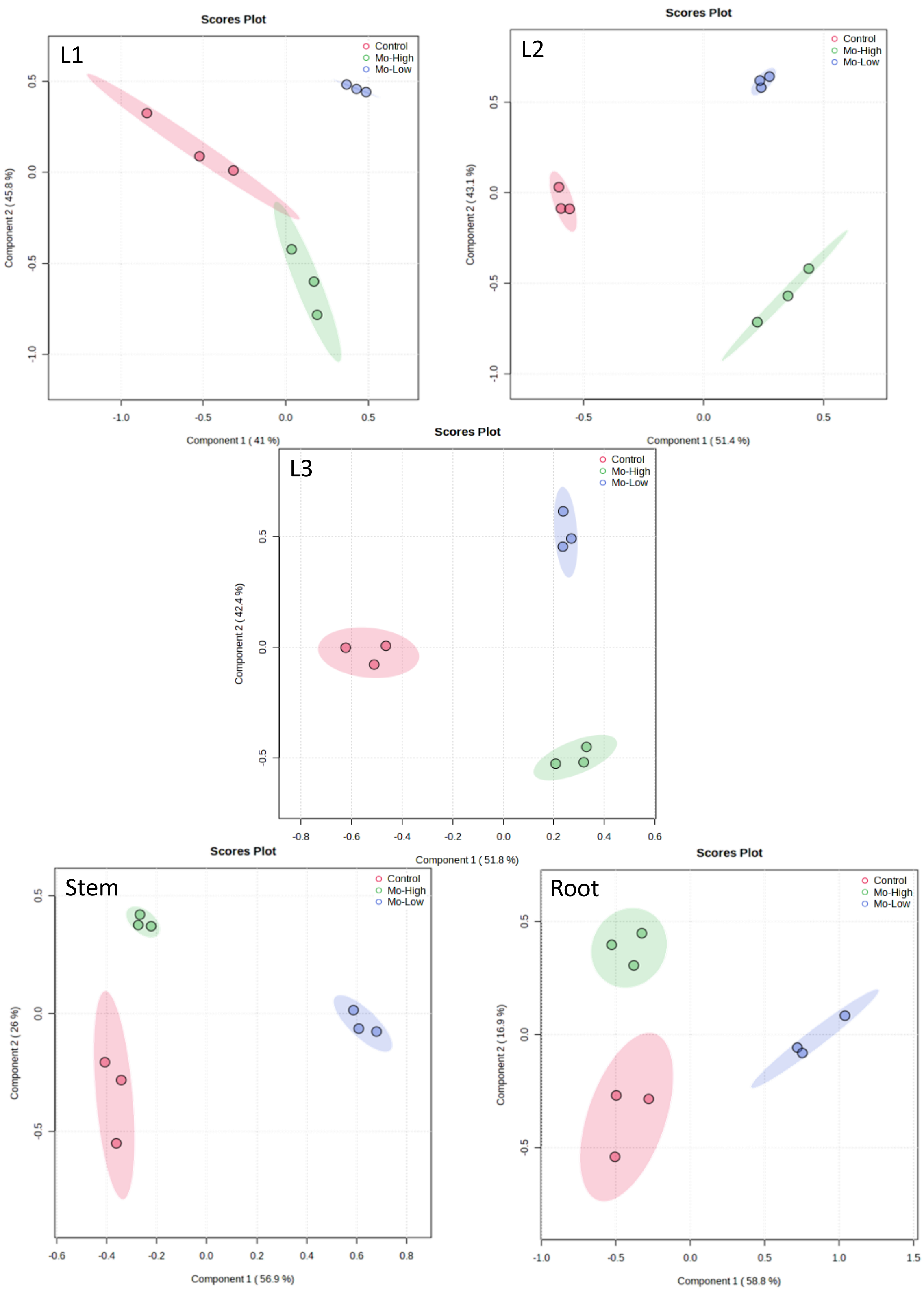

Figure S4. Partial Least Squares Discriminant Analysis (PLS-DA) of protein concentrations in different plant tissues exposed to high and low Mo doses via the roots.

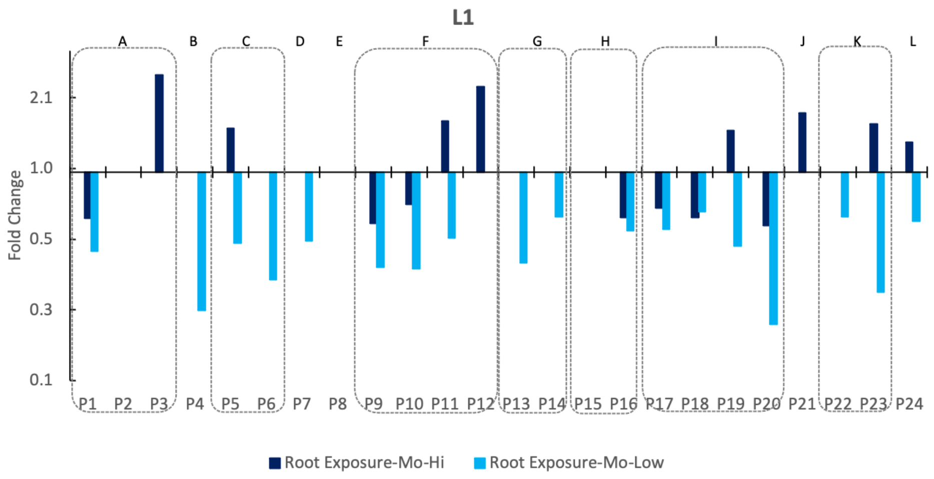
L1
A
B
C
D
E
F
G
H
I
J
K
L
Fold Change
2.1
1.0
0.5
0.3
0.1
P1 P2 P3 P4 P5 P6 P7 P8 P9 P10 P11 P12 P13 P14 P15 P16 P17 P18 P19 P20 P21 P22 P23 P24
Root Exposure-Mo-Hi
Root Exposure-Mo-Low

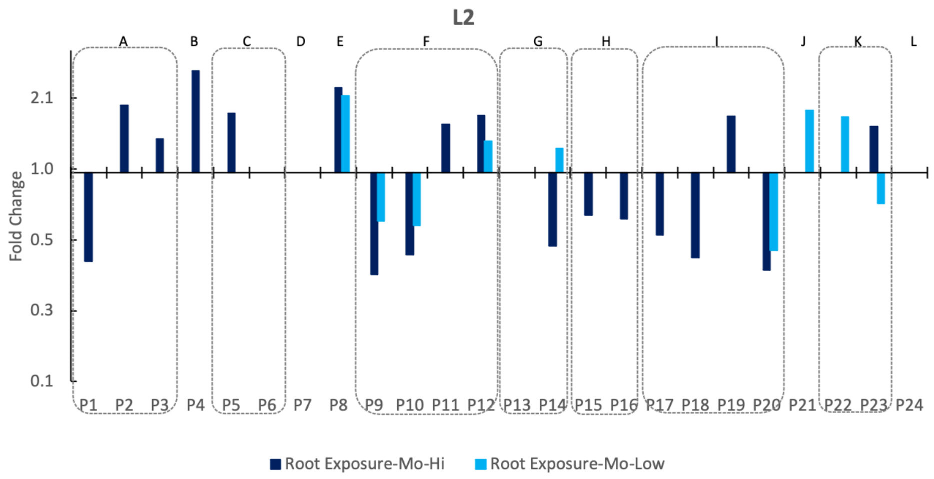
L2
A
B
C
D
E
F
G
H
I
J
K
L
Fold Change
2.1
1.0
0.5
0.3
0.1
P1 P2 P3 P4 P5 P6 P7 P8 P9 P10 P11 P12 P13 P14 P15 P16 P17 P18 P19 P20 P21 P22 P23 P24
Root Exposure-Mo-Hi
Root Exposure-Mo-Low

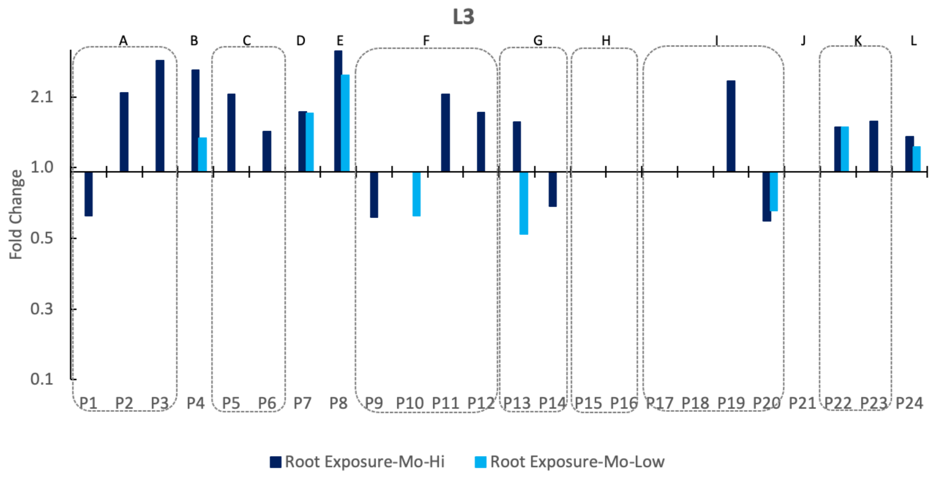
L3
A
B
C
D
E
F
G
H
I
J
K
L
Fold Change
2.1
1.0
0.5
0.3
0.1
P1 P2 P3 P4 P5 P6 P7 P8 P9 P10 P11 P12 P13 P14 P15 P16 P17 P18 P19 P20 P21 P22 P23 P24
Root Exposure-Mo-Hi
Root Exposure-Mo-Low

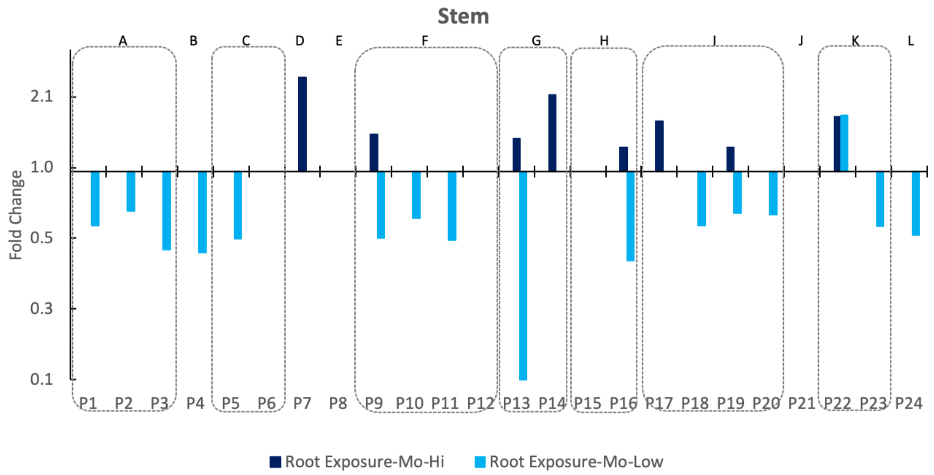

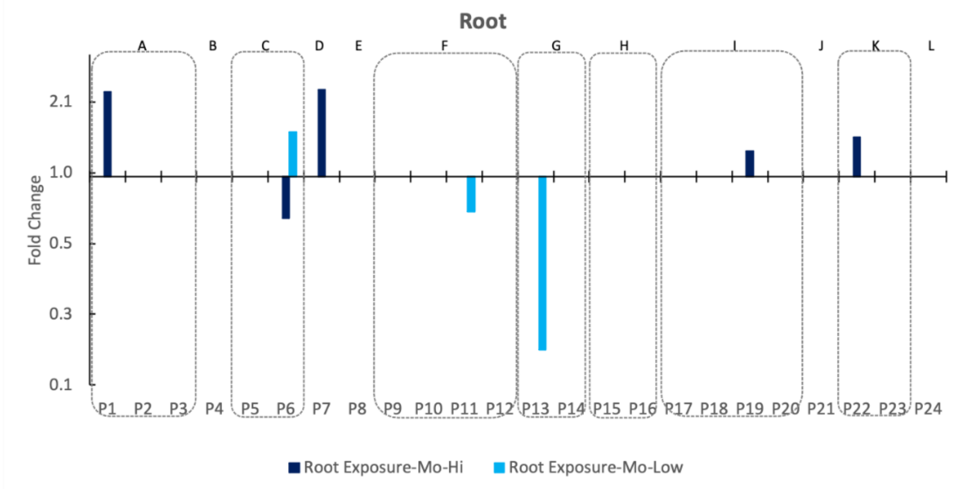

Figure S5. Fold change bar plots of proteins with FC ≥ 1.25 or ≤ 0.75 significant changes in different plant tissues with high and low Mo exposure treatments.

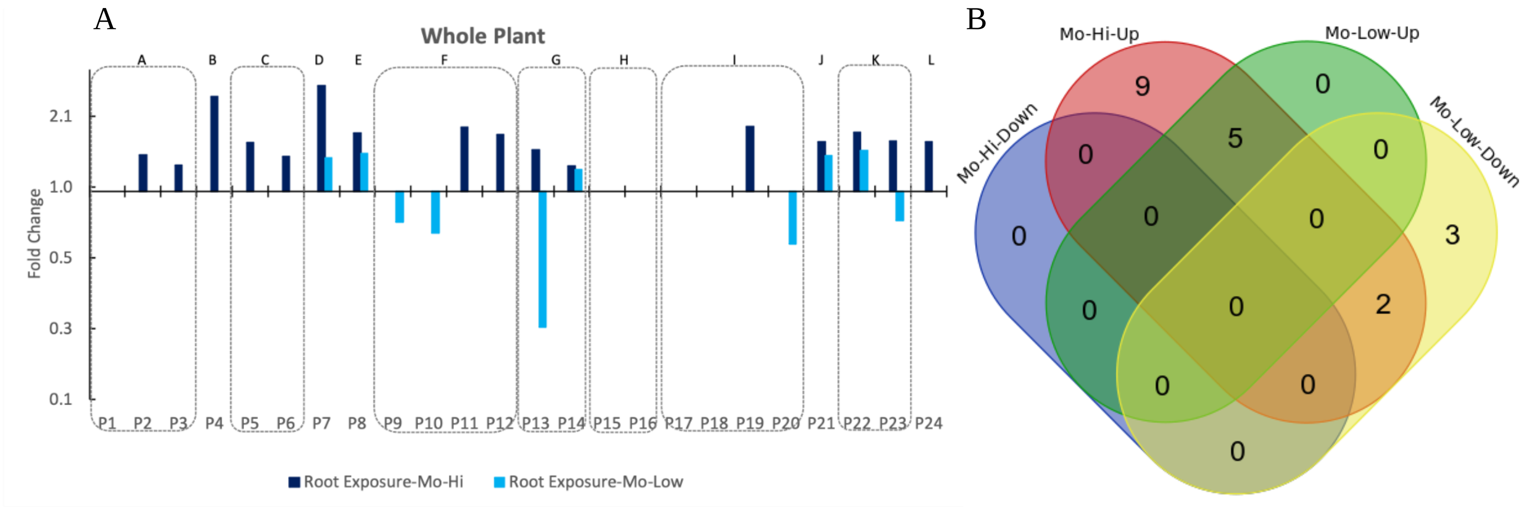

Figure S6. Protein expression in whole plant. A) Fold change bar plot of proteins with FC ≥ 1.25 or ≤ 0.75 significant changes in the whole plant with high and low Mo exposure treatments.; B) Venn diagram of proteins with FC ≥ 1.25 or ≤ 0.75 significant changes in the whole plant.
